# RV144 vaccine imprinting constrained HIV-1 evolution following breakthrough infection

**DOI:** 10.1093/ve/veab057

**Published:** 2021-07-09

**Authors:** Eric Lewitus, Eric Sanders-Buell, Meera Bose, Anne Marie O’Sullivan, Kultida Poltavee, Yifan Li, Hongjun Bai, Thembi Mdluli, Gina Donofrio, Bonnie Slike, Hong Zhao, Kim Wong, Lennie Chen, Shana Miller, Jenica Lee, Bahar Ahani, Steven Lepore, Sevan Muhammad, Rebecca Grande, Ursula Tran, Vincent Dussupt, Letzibeth Mendez-Rivera, Sorachai Nitayaphan, Jaranit Kaewkungwal, Punnee Pitisuttithum, Supachai Rerks-Ngarm, Robert J O’Connell, Holly Janes, Peter B Gilbert, Robert Gramzinski, Sandhya Vasan, Merlin L Robb, Nelson L Michael, Shelly J Krebs, Joshua T Herbeck, Paul T Edlefsen, James I Mullins, Jerome H Kim, Sodsai Tovanabutra, Morgane Rolland

**Affiliations:** US Military HIV Research Program, WRAIR, Silver Spring, MD 20910, USA; Henry M. Jackson Foundation for the Advancement of Military Medicine, Inc, Bethesda, MD 20817, USA; US Military HIV Research Program, WRAIR, Silver Spring, MD 20910, USA; Henry M. Jackson Foundation for the Advancement of Military Medicine, Inc, Bethesda, MD 20817, USA; US Military HIV Research Program, WRAIR, Silver Spring, MD 20910, USA; Henry M. Jackson Foundation for the Advancement of Military Medicine, Inc, Bethesda, MD 20817, USA; US Military HIV Research Program, WRAIR, Silver Spring, MD 20910, USA; Henry M. Jackson Foundation for the Advancement of Military Medicine, Inc, Bethesda, MD 20817, USA; US Military HIV Research Program, WRAIR, Silver Spring, MD 20910, USA; US Military HIV Research Program, WRAIR, Silver Spring, MD 20910, USA; Henry M. Jackson Foundation for the Advancement of Military Medicine, Inc, Bethesda, MD 20817, USA; US Military HIV Research Program, WRAIR, Silver Spring, MD 20910, USA; Henry M. Jackson Foundation for the Advancement of Military Medicine, Inc, Bethesda, MD 20817, USA; US Military HIV Research Program, WRAIR, Silver Spring, MD 20910, USA; Henry M. Jackson Foundation for the Advancement of Military Medicine, Inc, Bethesda, MD 20817, USA; US Military HIV Research Program, WRAIR, Silver Spring, MD 20910, USA; Henry M. Jackson Foundation for the Advancement of Military Medicine, Inc, Bethesda, MD 20817, USA; US Military HIV Research Program, WRAIR, Silver Spring, MD 20910, USA; Henry M. Jackson Foundation for the Advancement of Military Medicine, Inc, Bethesda, MD 20817, USA; Department of Microbiology, University of Washington, Seattle, WA 98195, USA; Department of Microbiology, University of Washington, Seattle, WA 98195, USA; Department of Microbiology, University of Washington, Seattle, WA 98195, USA; US Military HIV Research Program, WRAIR, Silver Spring, MD 20910, USA; Henry M. Jackson Foundation for the Advancement of Military Medicine, Inc, Bethesda, MD 20817, USA; US Military HIV Research Program, WRAIR, Silver Spring, MD 20910, USA; Henry M. Jackson Foundation for the Advancement of Military Medicine, Inc, Bethesda, MD 20817, USA; US Military HIV Research Program, WRAIR, Silver Spring, MD 20910, USA; Henry M. Jackson Foundation for the Advancement of Military Medicine, Inc, Bethesda, MD 20817, USA; US Military HIV Research Program, WRAIR, Silver Spring, MD 20910, USA; Henry M. Jackson Foundation for the Advancement of Military Medicine, Inc, Bethesda, MD 20817, USA; US Military HIV Research Program, WRAIR, Silver Spring, MD 20910, USA; Henry M. Jackson Foundation for the Advancement of Military Medicine, Inc, Bethesda, MD 20817, USA; US Military HIV Research Program, WRAIR, Silver Spring, MD 20910, USA; Henry M. Jackson Foundation for the Advancement of Military Medicine, Inc, Bethesda, MD 20817, USA; US Military HIV Research Program, WRAIR, Silver Spring, MD 20910, USA; Henry M. Jackson Foundation for the Advancement of Military Medicine, Inc, Bethesda, MD 20817, USA; US Military HIV Research Program, WRAIR, Silver Spring, MD 20910, USA; Henry M. Jackson Foundation for the Advancement of Military Medicine, Inc, Bethesda, MD 20817, USA; US Military HIV Research Program, WRAIR, Silver Spring, MD 20910, USA; Henry M. Jackson Foundation for the Advancement of Military Medicine, Inc, Bethesda, MD 20817, USA; US Army Medical Directorate of the Armed Forces Research Institute of Medical Sciences, Bangkok, Thailand; US Army Medical Directorate of the Armed Forces Research Institute of Medical Sciences, Bangkok, Thailand; Mahidol University, Nakhon Pathom, Thailand; Thai Ministry of Public Health, Nonthaburi, Thailand; US Army Medical Directorate of the Armed Forces Research Institute of Medical Sciences, Bangkok, Thailand; Fred Hutchinson Cancer Research Center, 1100 Fairview Ave. N., Seattle, WA 98109, USA; Fred Hutchinson Cancer Research Center, 1100 Fairview Ave. N., Seattle, WA 98109, USA; US Military HIV Research Program, WRAIR, Silver Spring, MD 20910, USA; US Military HIV Research Program, WRAIR, Silver Spring, MD 20910, USA; Henry M. Jackson Foundation for the Advancement of Military Medicine, Inc, Bethesda, MD 20817, USA; US Military HIV Research Program, WRAIR, Silver Spring, MD 20910, USA; Henry M. Jackson Foundation for the Advancement of Military Medicine, Inc, Bethesda, MD 20817, USA; Center for Infectious Disease Research, WRAIR, Silver Spring, MD 20910, USA; US Military HIV Research Program, WRAIR, Silver Spring, MD 20910, USA; Henry M. Jackson Foundation for the Advancement of Military Medicine, Inc, Bethesda, MD 20817, USA; Department of Global Health, University of Washington, Seattle, WA 98195, USA; Fred Hutchinson Cancer Research Center, 1100 Fairview Ave. N., Seattle, WA 98109, USA; Department of Microbiology, University of Washington, Seattle, WA 98195, USA; US Military HIV Research Program, WRAIR, Silver Spring, MD 20910, USA; US Military HIV Research Program, WRAIR, Silver Spring, MD 20910, USA; Henry M. Jackson Foundation for the Advancement of Military Medicine, Inc, Bethesda, MD 20817, USA; US Military HIV Research Program, WRAIR, Silver Spring, MD 20910, USA; Henry M. Jackson Foundation for the Advancement of Military Medicine, Inc, Bethesda, MD 20817, USA

**Keywords:** HIV-1, vaccine, within-host evolution, sieve analysis

## Abstract

The scale of the HIV-1 epidemic underscores the need for a vaccine. The multitude of circulating HIV-1 strains together with HIV-1’s high evolvability hints that HIV-1 could adapt to a future vaccine. Here, we wanted to investigate the effect of vaccination on the evolution of the virus post-breakthrough infection. We analyzed 2,635 HIV-1 *env* sequences sampled up to a year post-diagnosis from 110 vaccine and placebo participants who became infected in the RV144 vaccine efficacy trial. We showed that the Env signature sites that were previously identified to distinguish vaccine and placebo participants were maintained over time. In addition, fewer sites were under diversifying selection in the vaccine group than in the placebo group. These results indicate that HIV-1 would possibly adapt to a vaccine upon its roll-out.

## 1. Introduction

HIV-1 infection is typically established by a single variant (Keele et al., 2008), which diversifies rapidly through repeated, fragmentary selective sweeps driven by the host immune response (Alizon and Magnus 2012; Edwards et al., 2006; Shankarappa et al., 1999; Shriner et al., 2006; Wood et al., 2009). A high replication rate means that advantageous mutations become fixed quickly in the population (Wood et al., 2009), while deleterious mutations are purified out (Edwards et al., 2006). Viral diversification can occur rapidly in early HIV-1 infection: this viral adaptation to a new host environment attests to the fitness of HIV-1 upon transmission (Fraser et al., 2014). Taking into account the diversification patterns of HIV-1 is important to design vaccine candidates where specific viral features may present an evolutionary advantage over the host immune response.

Only one HIV-1 vaccine efficacy (VE) trial, RV144, has shown a reduction in the number of infections among trial participants (by 31 per cent, 95 per cent CI: 1.1 − 52.2 per cent, *P* = 0.04) (Rerks-Ngarm et al., 2009). A correlates study showed that the decreased risk of HIV-1 infection was associated with high Immunoglobulin G (IgG)-binding antibodies (Abs) against Env’s variable loops V1 and V2 (Haynes et al., 2012). Additional studies have sought to describe how a reduced risk of infection can be linked to V2-specific binding Abs rather than to more traditional correlates of vaccine protection such as neutralizing Ab responses or cytotoxic T lymphocyte (CTL) responses (Chung et al., 2014, 2015; Haynes et al., 2012; Zolla-Pazner et al., 2014). The importance of V2-specific responses was corroborated by sieve analyses, which compared sequences from vaccine and placebo recipients that became infected during VE trials. These analyses demonstrated that the RV144 vaccine (ALVAC-HIV and AIDSVAX B/E gp120) imposed an additional pressure on the virus as HIV-1 breakthrough sequences from vaccinees differed from those sampled in placebo recipients (Edlefsen et al., 2015; Rolland et al., 2012). We identified twelve sieve signature sites in Env; two sieve signatures were in V2, including at site 169 that has been associated with V2-specific Ab pressure in other cohorts (Moore et al., 2012). Similarly, the Step (Rollandet al., 2011) and HVTN 505 vaccine trials (deCamp et al., 2017), despite showing no overall clinical efficacy, were associated with differences between vaccine and placebo recipients in genes corresponding to the vaccine insert(s). Differences between vaccine and placebo groups were observed at HIV-1 diagnosis, and it is unclear whether these differences will be sustained over time. Would the evolutionary response in vaccinees manifest long-term consequences of vaccination? Or, would the Env sieve signatures revert over time?

Vaccine-elicited V2 binding responses declined rapidly in the 6 months after peak immunity (Montefiori et al., 2012; Robb et al., 2012; Yates et al., 2014), suggesting that vaccine pressure was transient. Thus, a potentially waning vaccine pressure together with HIV-1’s rapid capacity to mutate led us to hypothesize that changes may occur in the first few months after diagnosis. We analyzed 2,635 HIV-1 envelope (*env*) sequences derived via end point dilution from plasma samples collected from 110 RV144 participants who became HIV-1 infected during the trial (Edlefsen et al., 2015; Rolland et al., 2012). We showed that the Env sieve signatures identified at HIV-1 diagnosis were stable 6 months after diagnosis. In addition, we identified less viral diversification in vaccinees when compared to placebo recipients.

## 2. Materials and methods

### 2.1 Sequence analysis

A total of 2,635 *env* sequences were derived via single genome amplification from plasma samples collected from 110 individuals infected with HIV-1 CRF01_AE (as previously described (Rolland et al., 2012)). The data set included sequences from forty-four vaccine and sixty-six placebo recipients at diagnosis (Rolland et al., 2012) and twenty-eight vaccine and forty-five placebo recipients at 117–374 days post-diagnosis.

To ensure direct comparisons between sampling times, we conducted analyses on the twenty-eight vaccine and forty-five placebo recipients sampled at both diagnosis and post-diagnosis. Also, as analyses of alignments with more sampled sequences may be biased to detect more sites (Anisimova, Bielawski, and Yang 2002), we jackknifed the number of sequences, *n*_s_, for each individual by randomly removing *n*_s_ − *m*_s_ sequences, where *m*_s_ = the median number of sequences across participants, 100 times and analyzing all resulting sequence sets per participant. Median values were reported across jackknifed samples. This was done to ensure that the mean number of sequences per participant was the same across groups and sampling times. All primary analyses, unless otherwise stated, concerned jackknifed samples and included only participants sampled at both diagnosis and post-diagnosis. Where specified, analyses were run on all forty-four vaccine and sixty-six placebo recipients sampled at diagnosis.

Sequences were aligned with MAFFT v.7 (Katoh and Standley 2013) using an iterative refinement method incorporating local pairwise alignment information. Sequences were checked for hypermutation relative to HXB2 using Hypermut (https://www.hiv.lanl.gov/content/sequence/HYPERMUT/hypermut.html). Viral populations with multiplez founders, here called heterogeneous founder populations, were identified using a combination of qualitative and quantitative measures, as previously reported (Lewitus and Rolland 2019; Rolland et al., 2012; Rolland et al., 2020). Qualitative measures included the visual inspection of alignments, phylogenetic trees, highlighter plots, and plots of informative sites (https://indra.mullins.microbiol.washington.edu/DIVEIN/insites.html). Quantitative measures included pairwise diversity metrics (mean and maximum), the ratio of shared vs. private mutations, and a statistic based on the spectral density profile of the modified graph Laplacian. A set of independent HIV-1 CRF01_AE sequences collected during the same time period as RV144 infections occurred was downloaded from Los Alamos National Laboratory (LANL) (https://www.hiv.lanl.gov/components/sequence/HIV/search/search.html): a total of 521 unique Env sequences, sampled in 2004 (*n *= 49), 2005 (*n* = 43), 2006 (*n* = 65), and 2007 (*n* = 364) from individuals in Afghanistan (*n* = 1), Cameroon (*n* = 2), China (*n* = 305), Hong Kong (*n* = 1), Thailand (*n* = 211), and the United States (*n* = 1). These were aligned to HXB2 as above. No sequences from RV144 participants were included in this data set.

### 2.2 Amino acid mutations at specific sites

Amino acid variation was described by counting residues at twelve Env sites that had initially been identified as sieve signature sites that distinguished the vaccine and placebo groups (Edlefsen et al., 2015; Rolland et al., 2012). These sites were identified using methods designed to identify a sieve acquisition effect, with the Gilbert, Wu, and Jobes method (Gilbert, Wu, and Jobes 2008) as the primary approach. Using this method, all the participant’s sequences are represented by a single sequence (the sampled sequence closest to the consensus). While we consider that it is an acceptable approximation at HIV-1 diagnosis, we think that it is not appropriate to use a single sequence to summarize the diversity seen at 6 months post-diagnosis, leading us to not use sieve acquisition methods such as Gilbert, Wu, and Jobes at the second time point. The other approach we previously reported, the differential VE using the Lunn & McNeil method, was also not applicable at the second time point since it depends on the time to event. The number of unique sites under selection at each sampling time, as well as residue changes across sampling times at the twelve sites, was summarized for each treatment group. The same analyses were run by first dividing samples based on founder multiplicity (instead of treatment). For samples taken 6 months post-diagnosis, 95 per cent confidence intervals were computed across jackknifed samples.

At known Ab contact sites (*n* = 164), the consensus residue for each participant at diagnosis was compared with the residue in the CRF01_AE gp120 vaccine insert 92TH023. The consensus residue for each participant at diagnosis was then compared with the consensus at 6 months post-diagnosis. Additionally, to account for participants with fewer than the median number of sequences, any mutation at an Ab contact site that occurred in at least two sequences, but less than a majority of sequences, was substituted for the consensus in the above Ab contact site analysis. Only vaccinees that received four vaccinations were analyzed.

### 2.3 Prediction of CTL epitope repertoires

CTL epitopes were predicted using NetMHCPan4.1 (Reynisson et al., 2020) using a peptide length of 9 and selecting predicted peptides recognized as strong binders (binding affinity below 50 nM) from the output of NetMHCpan4.1. Predictions were made based on each individual’s combination of HIV-1 sequence and human leukocyte antigen (HLA) alleles.

### 2.4 Vaccine response (VR) score

A VR score was designed to integrate the twelve Env sieve signature sites associated with VE. Since we demonstrated genotype-specific VE in the RV144 trial, the score is based on the assumption that a vaccine would block sequences with specific residues at certain sites, and thus, sequences from vaccine recipients will show similar patterns of amino acids at specific sites due to vaccine escape by breakthrough strains. The effect can take two forms: (i) sites are more variable in vaccine compared to placebo recipients (e.g. site 169 in the RV144 cohort) and (ii) sites are less variable in vaccine compared to placebo recipients (e.g. site 181). The sequence of amino acids corresponding to the amino acids associated with VE (the VR_seq_) can, therefore, be formulated as amino acids that are statistically expected to be found disproportionately in vaccinees at specific sites. For RV144, the VR_seq_ is T6X, T19, K169, I181X, E268X, F317X, K343, F353, L369, R379X, T413X, and I424X. Therefore, due to breakthrough variants, a vaccinated individual is more likely to have a T at site 6, something other than a T at site 19, etc. A VR score can then be computed as follows:

1. A majority-rule consensus sequence is inferred for a participant at the sites of the VR_seq_. This sequence is called *P*_seq_ (Supplemental Fig. 4A).
2. Each site in *P*_seq_ is coded based on its consistency with the amino acid in the VR_seq_:
$$\begin{equation*}\Lambda (i) = \left\{ \begin{array}{c} {1\ {\text{if}}\ Pseq(i) \ne VRseq(i)} \\ {0\ {\text{otherwise}}}\end{array} \right.\end{equation*}$$
  For example, if the VR at site 19 is associated with T (T19), then the site in *P*_seq_ is counted as 0 if the amino acid is T and 1 if it is not T. Similarly, if the VR at site 181 is associated with the obverse of I (I181X), then the site at *P*_se__q_ is counted as 1 if the amino acid is I and 0 if it is not I (Supplemental Fig. 4B).
3. A hypothetical sequence is created for a participant in which amino acids at all the sites are consistent with blocking the VR_seq_ (e.g. T19 = Q19, I181X = I181, etc.). This is called *H*_seq_ (Supplemental Fig. 4C).
4. A probability density function (pdf) is computed for *H*_seq_. The median of the pdf is necessarily 1, but the interquartile range will depend on the length of *H*_seq_ (Supplemental Fig. 4D).
5. The position of the mean value of *P*_seq_, 0 ≤ *Λ_P_*_seq_ ≤ 1, is interpolated on the pdf of *H*_seq_ (Supplemental Fig. 4D).
6. The VR score for *P*_seq_ is calculated as one minus the distance between the interpolated point and the apex of the pdf (Supplemental Fig. 4D),
$$\begin{equation*} \Omega(x_1,x_2)= 1-\int_{x_1}^{x_2}\sqrt{1+(\frac{df}{dx})^2 dx} \end{equation*}$$

It is calculated as one minus the distance so that if all the amino acids in the *P*_seq_ are consistent with a VR blocking specific variants, then the VR score will be 1; whereas if there was no blockade from the VR (i.e. similar sequence as in the placebo group), the score will be 0. This provides an intuitive framework, whereby viruses with the most evidence that they escaped the vaccine pressure will have the highest VR scores.

Because the number of VR sites (*N*) is presumed to be a sample of the total number of actual sites associated with VE, a sample variance, *σ*^2^, is also calculated, where


$$\begin{equation*}{\sigma ^2} = {1 \over {N - 1}}\sum\nolimits_{i = 1}^N {{{({\text{z}}_{\text{i}}} - {\bar{\text{z}})}^2}} \end{equation*}$$

which can be used to account for the uncertainty in the estimate of the VR score.

To test the ability of this VR score to differentiate participants with different numbers of amino acids consistent with blocking the VR_seq_, we simulated nine 100 × twelve matrices with varying proportions (0 per cent − 100 per cent) of 1 s and 0 s. VR scores and sample variances were estimated for each matrix. VR scores could accurately differentiate between simulated participants with varying VEs (Supplemental Fig. 4E–G).

### 2.5 Estimates of diversification

Diversification dynamics were estimated on multiple sequence alignments for each participant using median and maximum pairwise distances within hosts, from the founder consensus estimated from the all-participant alignment at diagnosis and from the CRF01_AE consensus, 92TH023, CM244, and MN vaccine inserts. The founder consensus was based on the seventy-three participants sampled at both diagnosis and post-diagnosis, except when all 110 participants were analyzed, in which case it was based on all 110 participants. Pairwise distances were calculated using the dist.dna function in *ape* (Paradis and Schliep 2019) with substitution models inferred using ModelFinder (Kalyaanamoorthy et al., 2017).

Diversification patterns were compared across participants using a non-parametric phylogeny-based approach, which computes spectral density profiles for each phylogeny from their graph Laplacian and captures both the topology and the scale of the phylogenies (Lewitus and Morlon 2016; Lewitus and Rolland 2019). For each participant at each sampling point, unrooted, bifurcating phylogenetic trees were constructed from aligned sequences of *env* genes with IQ-TREE (Nguyen et al., 2015) assuming a substitution model inferred from ModelFinder (Kalyaanamoorthy et al., 2017). Breakpoints due to within-host recombination were tested using the Recombination Analysis Program (RAPR) (Song et al., 2018) against sequences for each participant at each sampling time and, if identified, used to partition alignments for tree reconstruction. Spectral density profile summary statistics (λ*, *ψ*, and *η*) were used to directly compare phylogenetic diversification between phylogenies and characterize their phylogenetic space (Lewitus and Morlon 2016). For a single sampling time, these statistics represent different aspects of the molecular rate of evolution, where λ* estimates the total distance traversed in the phylogeny, similar to phylogenetic diversity but accounting for the structure of the phylogeny, and is correlated with the non-synonymous/synonymous substitution rate, *ψ* estimates the skewness of branching patterns and reflects the heterogeneity of rates among branches, and *η* is an inverse of the transition-transversion rate ratio (Lewitus and Rolland 2019), which, in HIV-1, is shown to be a metric of mutational fitness (Lyons and Lauring 2017). We did not use gene-wide non-synonymous/synonymous rates, because these can lack power (Kryazhimskiy and Plotkin 2008) and produce erroneous results particularly among closely related sequences (Anisimova, Bielawski, and Yang 2002).

The number of purifying and diversifying sites at each sampling time was estimated using a fixed effects likelihood method on *env* genes that estimate non-synonymous and synonymous substitution rates at each codon (Kosakovsky Pond and Frost 2005). The number of sites was measured for alignments of all vaccine and all placebo recipients at each sampling time separately. Due to the higher number of sequences available for the placebo than vaccine group, the number of sequences in the placebo was jackknifed fifty times at the number of sequences in the vaccine group at each time point. The median value and 95 per cent confidence interval were computed on the number of purifying and diversifying sites across jackknifed alignments.

### 2.6 Hypervariable domain sequences per participant across sampling times

Sequences were divided into their hypervariable domains (V1–V5) using gene cutter (https://www.hiv.lanl.gov/content/sequence/GENE_CUTTER/cutter.html). Sequence diversity was compared between hypervariable domains using median and maximum pairwise distances. Comparisons were made between sampling times and treatment groups.

### 2.7 Analysis of *env* genes per participant across sampling times

Alignments made at each sampling time were separated per participant, and phylogenetic trees were constructed as above (Kalyaanamoorthy et al., 2017; Nguyen et al., 2015). Diversification dynamics were compared by sampling time, treatment group, and founder multiplicity using median and maximum pairwise distances (Paradis and Schliep 2019) and using spectral density profile summary statistics (Lewitus and Morlon 2016; Lewitus and Rolland 2019). A fixed effects likelihood method was used to estimate the number of sites per participant at each sampling time (Kosakovsky Pond and Frost 2005). Additional comparisons were made on neutralization breadth between treatment groups and between participants with homogeneous and heterogeneous founder populations.

### 2.8 Estimates of neutralization breadth

Neutralization assays were performed against thirty-four viruses using serum samples collected one (twenty-three vaccine and forty placebo recipients) and 3 years (thirty one vaccine and forty-nine placebo recipients) post-diagnosis (Mdluli et al., 2020). Analyses on neutralization breadth were performed on all participants with available data, although some were not included in the other analyses (because sequence data were not available at both sampling times). Neutralization breadth and the number of days since diagnosis were tested as predictors of the number of sites under selection and differences in diversification dynamics across participants.

### 2.9 Estimates of viral load and CD4 T cell counts

Set point viral load was calculated per participant as the average of pre-antiretroviral therapy log viral load measurements taken at any point in time between day 28 and day 365 post-infection diagnosis. Only participants who had more than two measurements contributing to the set point calculation were included. Set point viral load was available for twenty-four vaccine and fifty-seven placebo recipients.

## 3. Results

### 3.1 Data summary

We analyzed 994 *env* sequences (median of 10 ± 2.6 sequences per participant) previously obtained at HIV-1 diagnosis (Rolland et al., 2012) and derived 1,641 additional *env* sequences at a median of 186 days (range = 117 − 374) after diagnosis (Supplemental Fig. 1). The median number of days since diagnosis was 195 ± 47 for vaccine recipients and 183 ± 49 for placebo recipients, with no significant difference across groups (Student’s *t* test, *P* = 0.711). The original data set sampled at diagnosis included forty-four vaccine and sixty-six placebo recipients, all infected with CRF01_AE viruses. At the second sampling time (∼6 months post-diagnosis), sequences were available from twenty-eight vaccine and forty-five placebo recipients. The proportion of infections with heterogeneous founder populations, including multiple founders, and complex viral populations where diversification partially obscured the single/multiple classification was consistent across groups with thirteen (30 per cent) among vaccine recipients and twenty-two (33 per cent) among placebo recipients (Supplemental Fig. 1). Infections with heterogeneous or homogeneous founders were sampled at similar times from diagnosis (Student’s *t* test, *P* = 0.624 at diagnosis and *P* = 0.901 6 months later). Participants infected with heterogeneous founders showed higher viral load set point than those with homogeneous founder infections; however, there was no difference across treatment groups (Janes et al., 2015). There was no significant difference in markers of disease progression for vaccine and placebo recipients (Mdluli et al., 2020; Robb et al., 2012). For the subset of participants in our study, the median viral load set point was 4.57 RNA copies/ml in vaccine recipients and 4.42 RNA copies/ml for placebo participants (Mann–Whitney U test, *P* = 0.228). In addition, participants initiated antiretroviral treatment at similar times in vaccine (*n* = 28, median = 1,414 days post-diagnosis, min = 208, and max = 2,124) and placebo (*n* = 43, median = 1,502 days, min = 330, and max = 2,488) recipients (Student’s *t* test, *P* = 0.368).

### 3.2 Persistent signatures of vaccination at eleven Env sites

**Figure 1.**
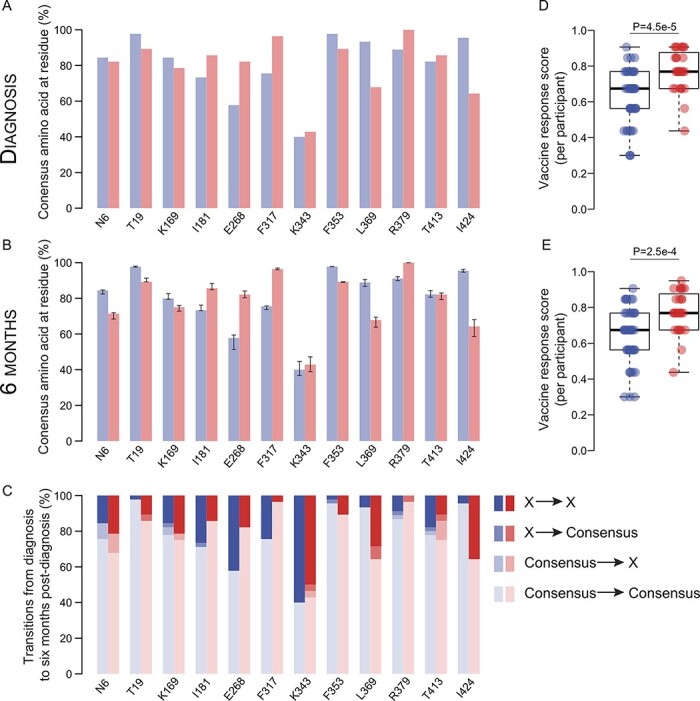
Sieve signatures distinguishing vaccine and placebo groups persisted over time. At twelve Env signature sites, the proportion of participants having the consensus amino acid is shown for vaccine (red) and placebo (blue) groups at (A) diagnosis and (B) 6 months post-diagnosis. Error bars in (B) indicate 95 per cent confidence intervals across jackknifed samples. (C) Proportion of transitions (median values across jackknifed samples) between consensus and non-consensus amino acids across sampling times in vaccine and placebo recipients. The per-participant VR scores corresponding to the twelve sieve signature sites that were calculated at (D) diagnosis and (E) 6 months post-diagnosis were significantly higher in vaccine recipients (*P*-values are shown for Mann–Whitney U *t* tests).

We previously showed that Env sequences differed significantly between vaccine and placebo recipients at twelve sites (Edlefsen et al., 2015; Rolland et al., 2012). At these twelve sieve signature sites, specific residues were associated with different VEs. Figure 1A shows the proportion of participants that had the consensus residue at each site. These data correspond to the subset of seventy-three participants with sequences available at two time points. When compared with the original set of 110 participants, the distribution of residues and the sign of the difference between vaccine and placebo recipients were concordant (Supplemental Fig. 2), showing that the subset of seventy-three participants is representative of the cohort. Because these twelve sieve signature sites distinguished the vaccine and placebo groups, we hypothesized that this restricted set of sites may be under stronger pressure to mutate than other Env sites. Yet, we found that eleven of these sieve signatures were maintained over time (Fig. 1B). Only site 413 showed that the consensus residue T was slightly more frequent among vaccinees than among placebo recipients at diagnosis, while the opposite was seen 6 months later; this visual assessment was supported by 95 per cent confidence intervals that overlapped at 6 months. In agreement with the maintenance of sieve signatures, a transition from consensus to non-consensus or vice versa was seen in only a small proportion of sites (2.4 per cent) (Fig. 1C). We compared the conservation of the sieve sites with their conservation in 521 CRF01_AE sequences sampled during the same time period. We found that the twelve signature sites corresponded to a range of variables to highly conserved HIV-1 Env sites in circulating sequences (consensus residues corresponding to frequencies of 31 per cent for K343 and 97 per cent for R379) (Supplementary Fig. 3).

To summarize the vaccine impact across these twelve sites, we developed a VR score, which ranks participants across sites based on the degree of escape from the vaccine (per-participant VR score, Supplemental Fig. 4). The per-participant VR scores were significantly higher in vaccine compared to placebo recipients, indicating that sequences in each vaccinated participant reflected escape patterns consistent with the VE results at both diagnosis (Fig. 1D) and 6 months later (Fig. 1E). These differences remained when only homogeneous or heterogeneous founders were compared at diagnosis (Mann–Whitney U test, *P* < 0.008) and 6 months later (*P* < 0.027).

### 3.3 Lack of vaccine/placebo differentiation at sites under potential host immune pressure

**Figure 2.**
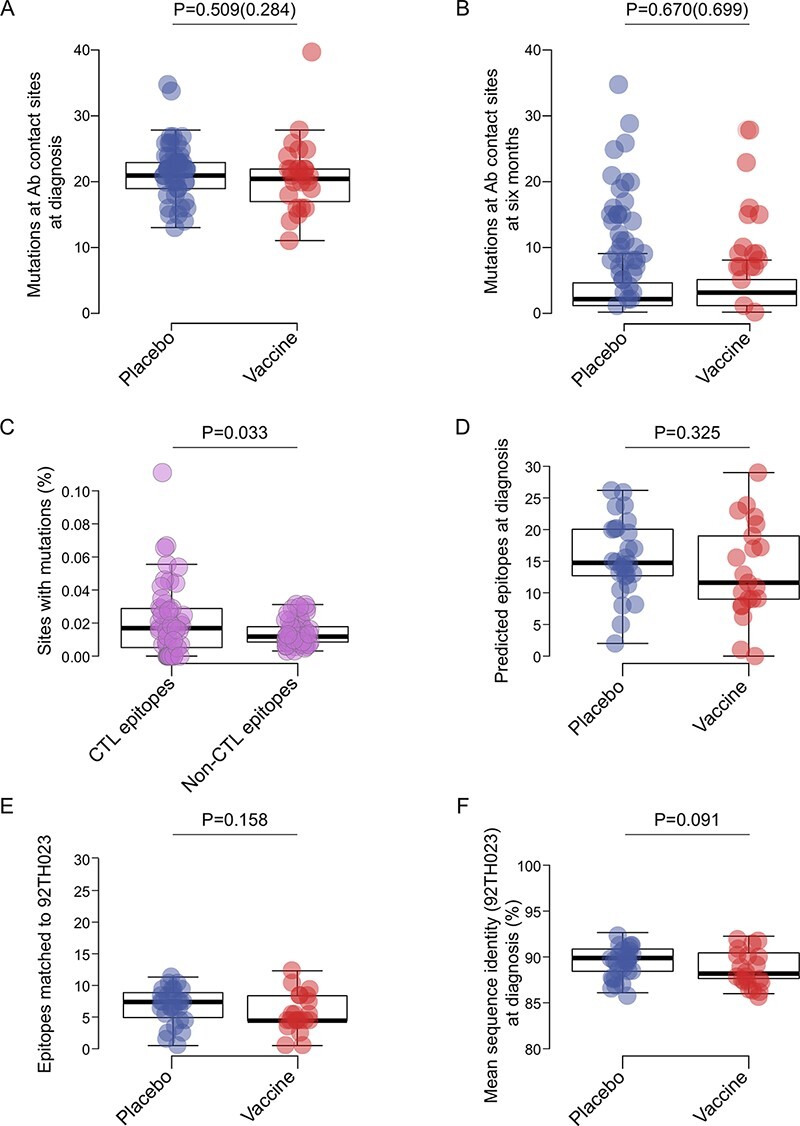
Mutations at sites associated with B or T cell pressure in Env did not differ across treatment groups. The number of mutations at known Ab contact sites (*n* = 164) per placebo (blue) and vaccine (red) participant at (A) diagnosis when comparing sequences with the CRF01_AE consensus and (B) 6 months post-diagnosis when comparing these sequences with those sampled at diagnosis. Swarm plots show the number of mutations after correcting for differences in the number of sequences per participant. (C) The proportion of sites with mutations considering sites known as CTL epitopes and sites without CTL epitopes (data from vaccine and placebo groups combined). (D) The number of CTL epitopes predicted on sequences sampled at diagnosis in vaccine and placebo recipients. (E) The number of CTL epitopes matching CRF01_AE gp120 vaccine insert 92TH023 in vaccine and placebo recipients at diagnosis. (F) Mean sequence identity between vaccine and placebo recipients and CRF01_AE gp120 vaccine insert 92TH023. *P*-values are shown for pairwise Student’s *t* tests (parenthetical *P*-values show results after correcting for the number of sequences in (a and b)).

To look beyond sites associated with VE in the RV144 trial, we analyzed 164 Env sites that have been identified as Ab contact sites in studies of natural HIV-1 infection (https://www.hiv.lanl.gov/components/sequence/HIV/featuredb/search/env_ab_search_pub.comp). At diagnosis, we found that an average of twenty-one sites showed mutations in both groups (Student’s *t* test, *P* = 0.509) (Fig. 2A). At 6 months, when sequences were compared with those sampled at diagnosis, we found an average of four sites that differed over time with no difference across groups (*P* = 0.670) (Fig. 2B).

We also evaluated sites that may be linked to CTL pressure by predicting CTL epitope repertoires for each individual (using each participant’s sequences and combination of HLA alleles). Mutations were significantly more frequent at sites associated with CTL epitopes than at other Env sites (*P* = 0.033) (Fig. 2C), as expected (Allen et al., 2005; Liu et al., 2006). However, similar numbers of CTL epitopes were predicted across groups: median values of 11.6 epitopes were identified in the vaccine group and 14.7 in the placebo group (*P* = 0.325) (Fig. 2D). The predicted number of epitopes did not vary over 6 months in either vaccine (*P* = 0.564) or placebo (*P* = 0.342) recipients. At HIV-1 diagnosis, the number of CTL epitopes matching the RV144 CRF01_AE gp120 vaccine insert 92TH023 tended to be lower in the vaccine group (median 4 epitopes predicted) than in the placebo group (seven epitopes) (*P* = 0.158) (Fig. 2E); similar trends were observed against the CM244 and MN vaccine inserts or CRF01_AE consensus, but differences were not significant (*P* ≥ 0.363, Supplemental Fig. 5A–C). This vaccine/placebo difference was driven by the fact that breakthrough sequences sampled from vaccinees tended to be more distant from the vaccine insert 92TH023 than those from placebo recipients (*P* = 0.091) (Fig. 2F); similar trends were observed against the CM244 and MN vaccine inserts or CRF01_AE consensus, but differences were not significant (*P* ≥ 0.416, Supplemental Fig. 5D–F). This reflected a founder effect associated with differential HIV-1 variant acquisition following vaccination. There was no change between diagnosis and 6 months later in vaccine (Wilcoxon signed-rank test, *P* = 0.564) or placebo (*P* = 0.342) recipients.

### 3.4 Increase in the number of minor variants at signature sites in placebo but not in vaccine recipients

**Figure 3.**
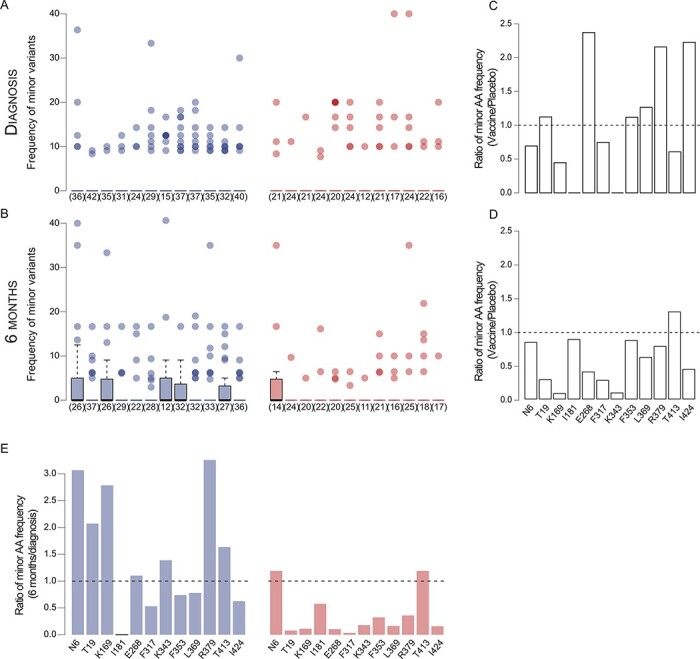
Minor variants increased at sieve signature sites in placebo but not in vaccine recipients. The percentage represented by non-consensus amino acids in vaccine (red) and placebo (blue) recipients at each signature site at (A) diagnosis and (B) 6 months post-diagnosis. This is calculated for infections where the consensus corresponding to the signature site is found in the majority of sequences from that individual; the number of participants with zero values is noted in parentheses. Median values across jackknifed samples are shown in (B). The ratio of the mean number of non-consensus (minor) amino acids per consensus-majority participant in vaccine and placebo recipients at (C) diagnosis and (D) 6 months post-diagnosis at each Env signature site. (C and D) Dashed lines indicate an equal number of non-consensus amino acids between vaccine and placebo recipients; values above the dashed line indicate a higher number in vaccinees. (E) The mean ratio of non-consensus amino acid frequencies at each signature site across sampling times in placebo and vaccine recipients. The dashed line indicates the value if there was no difference in frequencies across sampling times; values above the dashed line indicate an increase in non-consensus amino acids between diagnosis and 6 months post-diagnosis.

We evaluated whether vaccination had an effect on HIV-1 diversification. In individuals infected with HIV-1, rare mutations usually increase in frequency over time. As such, among placebo recipients, there was a median of 7.5 signature sites with mutations at 6 months post-diagnosis as opposed to three such mutating sites at diagnosis. However, this was not observed for vaccinees, with median values of 2 and 2.5 signature sites with variation at diagnosis and 6 months, respectively (Fig. 3A–B). Across the twelve signature sites, the median ratio of the frequency of non-consensus amino acids (or minor variants) in vaccine (0.176) over placebo (0.162) recipients was 1.1 at diagnosis (Fig. 3C) and 0.35 6 months later (Fig. 3D). On average, the frequency of non-consensus amino acids or minor variants increased by 1.8 fold (± 0.52 SE) in placebo recipients, while it decreased by almost 3 fold (± 0.35 SE) in vaccinees (Fig. 3E).

We then compared the maximum pairwise sequence diversity (Supplemental Fig. 6A) and patterns of phylogenetic diversification (Supplemental Fig. 6B–D). We found limited evidence of recombination: at diagnosis, 2.2 per cent (1/44) of vaccine recipients and 4.5 per cent (3/66) of placebo recipients showed evidence of recombination, all of which had multiple founders; at 6 months post-diagnosis, 10.1 per cent (3/28) of vaccine recipients, 66.7 per cent with multiple founders, and 24.4 per cent (11/45) of placebo recipients, 72.7 per cent with multiple founders, showed evidence of recombination. The total distance traversed in the phylogenetic network, estimated by mean λ* values, was significantly lower in vaccinees compared to placebo recipients after 6 months (*P* = 0.011) (Supplemental Fig. 6D). There was a significant negative correlation between λ* and per-participant VR scores at 6 months post-diagnosis (*R*^2^ = 0.129, *P* = 0.002) but not at diagnosis (*R*^2^ = 0.037, *P* = 0.065) (Supplemental Fig. 6E). Although the subset of infections with heterogeneous founder populations had significantly higher maximum sequence diversity (or λ*) than those with homogeneous founders, this did not drive differences across treatment groups. Hence, when only participants with homogeneous founders were compared, vaccinees still had significantly lower λ* than placebo recipients at 6 months post-diagnosis (Mann–Whitney U test, *P* = 0.038, Supplemental Fig. 7). There was a weak but significant relationship between the λ* and the heterogeneity of branch lengths (*ψ*) among placebo (*R*^2^ = 0.178, *P* = 2.303 × 10^−5^) but not among vaccine (*R*^2^ = 0.079, *P* = 0.061) recipients (Supplemental Fig. 8). This suggests that the total phylogenetic diversity λ*, which is a correlative of the strength of selection, in placebo recipients was driven, in part, by an unequal distribution of variance across lineages.

We also compared diversification dynamics separately for each Env hypervariable domain. At diagnosis, we found slightly higher maximum sequence diversity in placebo compared to vaccine recipients in Env-V1 (*P* = 0.010) (Supplemental Fig. 9A). As expected, over 6 months, intra-host maximum sequence diversity increased in all hypervariable domains. The mean fold increase was smaller in vaccine recipients (1.7, SD = 0.54) than in placebo recipients (2.3, SD = 0.52), yet the difference was not significant (Supplemental Fig. 9B).

### 3.5 Lower diversifying selection in vaccinees at 6 months post-infection

**Figure 4.**
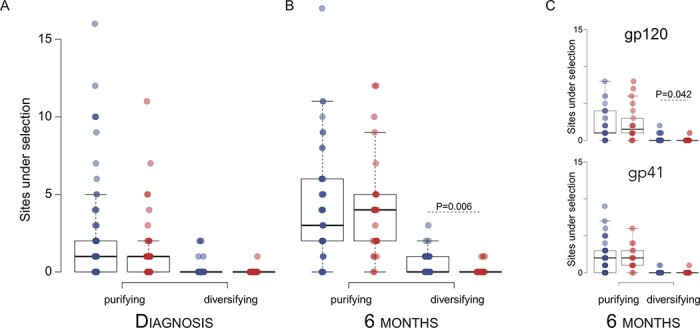
Diversifying selection observed in placebo but not vaccine recipients. The number of sites under purifying and diversifying selection per participant is shown for vaccine (red) and placebo (blue) recipients at (A) diagnosis and (B) 6 months post-diagnosis. (C) Per-participant sites under selection at 6 months post-diagnosis in Env-gp120 and Env-gp41. Significant pairwise differences are indicated with the associated *P*-value (Student’s *t* test).

We identified Env sites that were under purifying or diversifying selection among sequences from each individual. Per participant, the number of purifying or diversifying sites identified at diagnosis did not differ across groups (Mann–Whitney U test, *P* > 0.11) (Fig. 4A). 6 months after diagnosis, sequences from vaccinees showed significantly fewer sites under diversifying selection than placebo recipients (*P* = 0.006) (Fig. 4B). Hence, there was a significant increase in diversifying sites over time only in placebo recipients (mean *t*_1_ = 0.12, mean *t*_2_ = 0.45; *P* = 0.008, Hedge’s *G* = 0.58), not in vaccine recipients (mean *t*_1_ = 0.03, mean *t*_2_ = 0.11; *P* = 0.220, Hedge’s *G* = 0.37). This vaccine/placebo difference was linked to gp120 (*P* = 0.042), the part of Env that was included in the vaccine and not seen in gp41 (*P* = 0.611) (Fig. 4C). This was not driven by differences due to infections with heterogeneous founders. As such, at diagnosis, there were no significant vaccine/placebo differences in the number of purifying or diversifying sites under selection when only infections with either homogeneous (Mann–Whitney U test, *P* ≥ 0.788) or heterogeneous (*P* ≥ 0.089) founders were considered (Supplemental Fig. 10A–B). 6 months later, there remained no vaccine/placebo difference in the number of purifying sites when only infections with either homogeneous (*P* = 0.363) or heterogeneous (*P* = 0.813) founders were considered (Supplemental Fig. 10C). However, there were significantly more sites under diversifying selection among sequences from placebo recipients than among vaccinees regardless of the homogeneous (*P* = 0.049) or heterogeneous (*P* = 0.005) founder status (Supplemental Fig. 10D).

Comparisons of the sites under selection across groups did not reveal that specific sites were targeted differentially: there was no difference in the median number of known Ab contact sites under selection per participant between vaccine and placebo recipients at diagnosis (Mann–Whitney U test, *P* = 0.882) or 6 months post-diagnosis (*P* = 0.811). Days since diagnosis was also not a significant predictor of the number of sites under selection (*R*^2^ < 0.040, *P* > 0.798).

The analysis of sequences across all participants yielded similar results. Among vaccine recipients, there were significantly fewer unique sites under purifying selection at both diagnosis (Pearson’s *χ*-squared, *P* = 0.036) and post-diagnosis (*P* = 3.709 × 10^−7^) when compared to sequences from placebo participants. Likewise, for diversifying selection, sequences from vaccinees showed fewer sites under diversifying selection at diagnosis (*P* = 0.036) and post-diagnosis (*P* = 0.012) compared to placebo recipients (Supplemental Fig. 11A–D). When the number of sequences for placebo recipients (393 at diagnosis; 507 at post-diagnosis) was downsampled to the number of vaccine recipients (230 at diagnosis; 488 at post-diagnosis), these differences remained significant (*P* < 0.047). At both diagnosis and 6 months after, the majority (> 62 per cent) of all sites under selection in vaccine recipients were also under selection in placebo recipients. When we compared how the sites under selection were distributed across Env, we found no significant difference between vaccine and placebo groups at diagnosis (Pearson’s *χ*-squared, *P* > 0.965) or post-diagnosis (*P* > 0.978), with about two thirds of the sites under selection found in gp120. There were no significant vaccine/placebo differences in the proportion of sites under selection for specific Ab targets (CD4bs, V1-V2 glycan, V3 glycan, or MPER) at diagnosis (*P* > 0.834) or 6 months post-diagnosis (*P* > 0.841) (Supplemental Fig. 11E–H).

### 3.6 Vaccination inhibited the development of neutralization breadth

**Figure 5.**
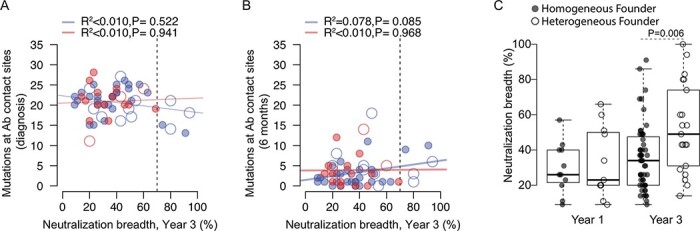
The lack of neutralization breadth in vaccine recipients was not associated with the number of potential escape mutations at Ab contact sites. Scatterplots of the neutralization breadth (measured 3 years after diagnosis) against the number of mutations at Ab contact sites in sequences from vaccine (*n* = 18, red) and placebo (*n* = 39, blue) recipients. Dashed lines indicate the threshold for broad neutralization breadth (70 per cent of viruses). Mutations are scored based on (A) sequences sampled at diagnosis compared to the CRF01_AE consensus and (B) sequences sampled 6 months later and compared to those sampled at diagnosis. Participants with homogeneous founder populations are shown with closed circles, and those with heterogeneous founders with open circles. Best-fit linear regressions are shown (solid lines) along with *R*^2^ and *P*-values. (C) Boxplot of neutralization breadth measured 1 year and 3 years post-diagnosis for participants infected with homogeneous or heterogeneous founders. A significant pairwise difference seen with neutralization data obtained 3 years post-diagnosis is indicated with the associated *P*-value (Student’s *t* test).

We previously showed that, 3 years after infection, vaccine recipients were less likely to develop neutralizing Ab responses than placebo recipients (Mann–Whitney U test, *P* = 0.033) (Mdluli et al., 2020). At Year 3, eight placebo recipients neutralized > 70 per cent of viruses in a panel of thirty-four viruses, while none of the vaccinees showed such a breadth. We found a weak relationship between HIV-1 diversification and neutralization breadth in placebo recipients: at Year 1, a higher number of purifying (*R*^2^ = 0.122, *P* = 0.040) and diversifying (*R*^2^ = 0.189, *P* = 0.013) sites under selection were associated with neutralization breadth (Supplemental Fig. 12A). At Year 3, only the association with diversifying sites (*R*^2^ = 0.101, *P* = 0.047) remained (Supplemental Fig. 12B). As described above, vaccine and placebo recipients had similar numbers of mutations at Ab contact sites (*P* > 0.284). We found that the number of mutations at Ab contact sites were not associated with neutralization breadth in vaccine or placebo recipients at diagnosis (*R*^2^ < 0.009, *P* ≥ 0.522) (Fig. 5A) or 6 months later (*R*^2^ ≤ 0.078, *P* ≥ 0.085) (Fig. 5B). These results suggest that the lack of development of neutralization breadth in vaccinees is not due to the differential presence of Ab escape mutations in sequences from vaccinees.

Founder multiplicity had a significant effect on the development of neutralization breadth (Fig. 5C), yet we did not see a relationship between the number of purifying or diversifying sites and neutralization breadth in either vaccine or placebo recipients when homogeneous or heterogeneous founders were analyzed separately (*R*^2^ < 0.098, *P* > 0.503) (possibly due to small group sizes).

## 4. Discussion

In the RV144 trial, the VE decreased from 59.9 per cent at 1 year to 31.2 per cent at 3.5 years (Karnasuta et al., 2017; Montefiori et al., 2012). Concurrently, vaccine-induced titers of binding Abs targeting V1V2, which correlated with a decreased risk of HIV-1 infection, declined over time (Montefiori et al., 2012; Robb et al., 2012; Yates et al., 2014). After responses peaked two weeks after the last immunization, V1V2 binding Abs waned rapidly over time and were usually not found 6 months after vaccination in individuals who became infected (Montefiori et al., 2012; Robb et al., 2012; Yates et al., 2014). For this reason, it was possible that sieve mutations would have reverted over time (in a process analogous to CTL escape mutations transmitted to an HLA-mismatched host). We found, however, that sieve signatures persisted over the first 6 months after diagnosis and that, more broadly, vaccination modified the evolutionary process for HIV-1 breakthrough infections.

These findings are the first evidence that a vaccine can disrupt HIV-1 evolution. Our results highlight the possibility of HIV-1 adaptation to a vaccine that would be distributed on a large scale, because the maintenance of sieve signatures over time forms the molecular basis for vaccine-induced HIV-1 strain replacement. Our results suggest that, upon roll-out of a partially efficacious vaccine, HIV-1 vaccine-escaped variants could become dominant in the population as time progresses and vaccine coverage increases. Understanding whether vaccination can impact HIV-1 evolution post-breakthrough infection is important because first-generation HIV-1 vaccines will most likely not eradicate all infections. Instead, these first-generation vaccines will likely reduce acquisition rates for a subset of HIV-1 strains, thereby possibly facilitating the emergence of new variant strains that will carry mutations such as the sieve signatures we identified.

Interestingly, in our study, sieve mutations were stable over time and did not revert in the first few months of HIV-1 infection as previously observed for CTL escape mutations (Carlson et al., 2016). These results indicate that sieve mutations were not disadvantageous and did not carry a significant fitness cost even as vaccine-specific Abs were no longer present in vaccine recipients, as exemplified by their varying degrees of sequence conservation in independent circulating CRF01_AE sequences. Such results may be linked to the fact that sieve signatures were identified in Env, where mutations typically carry less cost than mutations in more conserved HIV-1 Env proteins such as Gag. By modeling the roll-out of an HIV-1 vaccine with differential efficacy against specific genotypes, we previously showed that mutations associated with vaccine resistance would spread in the HIV-1 population, thereby reducing the overall effectiveness of the vaccine over time (Herbeck et al., 2018). Such vaccine-driven evolution has been studied for other pathogens. The introduction of the 7- and 13-valent pneumococcal conjugate vaccines was associated with a rapid serotype diversification and previously rare serotypes that caused invasive pneumococcal disease in children (Stockmann et al., 2016). Similarly, the switch from a whole-cell vaccine against Bordetella pertussis to a less reactogenic acellular vaccine led to the rapid emergence of new strains with alleles not included in the acellular vaccine (Bart et al., 2014).

While it was remarkable that the differences seen in Env at HIV-1 diagnosis did not revert in the 6 months after diagnosis, it was even more surprising that the diversification process in vaccinees lagged behind what was observed in placebo recipients. In the context of a randomized placebo controlled trial, differences between vaccine and placebo can be attributed to the vaccination. Although the effect of vaccine priming is more difficult to ascertain at later times in the infection given the known impact of host-induced immune responses on HIV-1 evolution (Goulder et al., 1997; Price et al., 1997; Richman et al., 2003), the maintenance of Env sieve signatures further supports our initial study that emphasized a sieve acquisition effect (akin to a founder effect) with RV144 VE associated with genotype-specific differences. It is complicated to separate sieve post-acquisition effects from host-induced immunity. To potentially disentangle these effects, we evaluated evidence of Ab and CTL-mediated immune pressure. Our results suggest that vaccine priming and the differences seen at HIV-1 diagnosis dictated early evolution in vaccine versus placebo participants. Both viral diversity and neutralization breadth are HIV-1 features that increase over the course of natural infection. HIV-1 diversity has been associated with the subsequent development of neutralization breadth (Landais et al., 2016; Moore et al., 2012; Sather et al., 2014). Thus, the higher diversity seen in placebo participants could have promoted the development of neutralization breadth in placebo recipients, while the development of neutralization breadth was hindered by the limited early diversification seen in vaccine recipients (Luo and Perelson 2015; Nourmohammad, Otwinowski, and Plotkin 2016). It is also demonstrated that neutralizing Abs drive viral evolution in an individual through repeated escape cycles (Liao et al., 2013; Richman et al., 2003). In this cohort, we know that vaccinees were primed to induce Ab responses. However, RV144 vaccination did not yield broadly neutralizing responses nor robust CTL responses. In contrast, the RV144 vaccine elicited binding Abs that were associated with a reduced risk of infection (Haynes et al., 2012; Mdluli et al., 2020). Binding (non-neutralizing) Abs and the Fc effector functions they mediate have not been linked with driving Env escape. Hence, the limited diversification seen in vaccinees is consistent with binding (and non-neutralizing) Abs and a vaccine-induced immune response geared toward Fc-mediated effector functions at the expense of the development of broad neutralization. The persistence of purifying selection in vaccinees at levels congruous with placebo recipients, which is a hallmark of low-frequency polymorphisms in *env* (Edwards et al., 2006), further supports the effect of vaccine priming on inhibiting the ability of the virus to diversify over time.

The constraining effect of the RV144 vaccine priming on HIV-1 diversification underscores the potential of vaccine strategies that seek to have disease-modifying benefits in the event of a breakthrough. As such, our data lend credence to vaccine designs that aim to ‘corner’ the virus to potentially attenuated forms in order to delay disease progression and reduce transmissibility (Altfeld and Allen 2006; Carlson et al., 2008; Gaiha et al., 2019; Letourneau et al., 2007; Mann and Ndung’u 2015; Rolland, Nickle, and Mullins 2007), effectively forcing the virus into an adaptive wasteland. The finding of post-infection differences in the diversification of HIV-1 in vaccinees has implications for future HIV-1 vaccines as first-generation HIV-1 vaccines will likely be only partially effective. A partially effective vaccine means that breakthrough variants could potentially come to dominate the viral population.

One limitation of our study is that we describe a unique scenario where an HIV-1 vaccine afforded modest protection against HIV-1, and we have no possibility to contrast our findings with other studies. The lack of efficacy observed in the HVTN 702 trial is another reminder that the search for an efficacious HIV vaccine is arduous. The HVTN 702 Phase III trial was designed as a follow-up to RV144, yet several variables differed between the RV144 and HVTN 702 trials. The vaccine insert, adjuvant, expected coverage of circulating viruses and the study population were different, and there was also an extra boost. The mechanisms behind the lack of efficacy in HVTN 702 need to be investigated, and our work can provide hypotheses to test.

This work has implications for HIV-1 vaccine research. The maintenance of sieve signatures highlights the possibility of vaccine-driven HIV-1 adaptation, whereby strains not included in the vaccine or resistant to the vaccine will outcompete other genotypes. Consequently, the vaccine may open new ecological niches that could be invaded by non-vaccine genotypes. Hence, our results highlight the potential risk of development of vaccine resistance, while also illustrating that vaccines could inhibit HIV-1 diversification, thereby suggesting renewed optimism for vaccine strategies that could drive HIV-1 to less virulent forms. These results emphasize that vaccine strategies will need to take into account HIV-1 evolutionary dynamics. By analogy to the emergence of HIV-1 drug resistance mutations that was abated by tri-therapies, we theorize that multi-pronged vaccine approaches would be needed to lessen the risk of HIV-1 vaccine resistance.

## Supplementary Material

veab057_Supp

## Data Availability

Sequences are available in GenBank under accession numbers MZ346605-MZ348228, along with previously generated sequences (JX446645-JX448316). Sequence alignments are available at https://www.hivresearch.org/publication-supplements.
