# Supplementary material for: RV144 vaccine imprinting constrained HIV-1 evolution following breakthrough infection
Source: Virus Evol. 2021 Jul 9;7(2):veab057. doi: 10.1093/ve/veab057 (PMC8438874; doi:10.1093/ve/veab057)
Supplement: veab057_Supp [file veab057_supp.zip › RV144.Sievebis_Supplement.20may2021.docx]

**Supplement**

**RV144 vaccine imprinting constrained HIV-1 evolution following breakthrough infection**

Eric Lewitus1,2, Eric Sanders-Buell1,2, Meera Bose1,2, Anne Marie O’Sullivan1,2, Kultida Poltavee1, Yifan Li1,2, Hongjun Bai1,2, Thembi Mdluli1,2, Gina Donofrio1,2, Bonnie Slike1,2, Hong Zhao3, Kim Wong, Lennie Chen3, Shana Miller1,2, Jenica Lee1,2, Bahar Ahani1,2, Steven Lepore1,2, Sevan Muhammad1,2, Rebecca Grande1,2, Ursula Tran1,2, Vincent Dussupt1,2, Letzibeth Mendez-Rivera1,2, Sorachai Nitayaphan4, Jaranit Kaewkungwal4, Punnee Pitisuttithum5, Supachai Rerks-Ngarm6, Robert J. O’Connell4, Holly Janes7, Peter Gilbert7, Robert Gramzinski1, Sandhya Vasan1,2, Merlin L Robb1,2, Nelson L Michael8, Shelly Krebs1,2, Joshua T. Herbeck9, Paul T. Edlefsen7, James I. Mullins3, Jerome H Kim1, Sodsai Tovanabutra1,2, Morgane Rolland1,2*

1US Military HIV Research Program, WRAIR, Silver Spring, Maryland, USA

2Henry M. Jackson Foundation for the Advancement of Military Medicine, Inc., Bethesda, Maryland, USA 3Department of Microbiology, University of Washington, Seattle, Washington, USA

4US Army Medical Directorate of the Armed Forces Research Institute of Medical Sciences, Bangkok, Thailand

5Mahidol University, Bangkok, Thailand
6Thai Ministry of Public Health, Nonthaburi, Thailand
7Fred Hutchinson Cancer Research Center, 1100 Fairview Ave. N., Seattle, WA 98109

8Center for Infectious Disease Research, WRAIR, Silver Spring, Maryland, USA

9Department of Global Health, University of Washington, Seattle, Washington, USA

*correspondence: mrolland@hivresearch.org

**Contents**

S1 Number of participants and sequences at diagnosis and post-diagnosis 2

S2 Frequency of CRF01_AE consensus and non-consensus amino acids at sieve sites for all (110) participants at diagnosis 3

S3 Amino acid frequencies at sieve sites in circulating CRF01_AE sequences 4

S4 Schematic of estimating a per-participant Vaccine Response (VR) score 5

S5 Mutations at sites associated with T cell pressure 6

S6 Temporal changes in diversification 7

S7 The effect of founder heterogeneity on l* 8

S8 Phylogenetic space of vaccine and placebo participants at diagnosis and six months later 9

S9 Temporal changes in sequence diversity across hypervariable domains 10

S10 The effect of founder heterogeneity on pervasive selection 11

S11 Per-site pervasive selection 12

S12 The effect of neutralization breadth on pervasive selection 13

**S1 Number of participants and sequences at diagnosis and post-diagnosis**


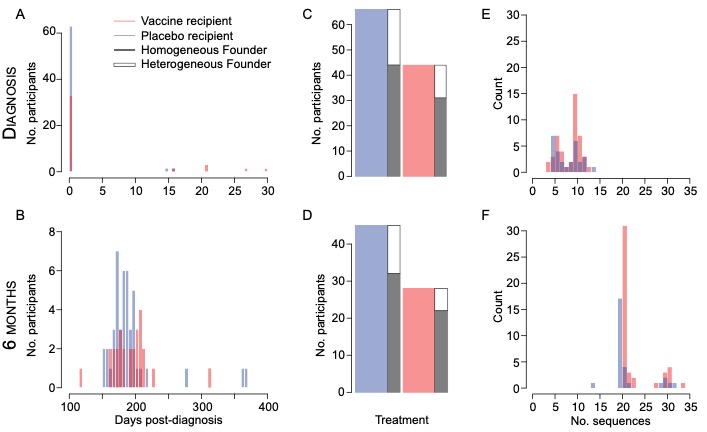


**Figure S1: Number of participants and sequences at diagnosis and post-diagnosis.** The number of participants with samples collected at a given time after diagnosis is shown at (A) diagnosis and (B) approximately six months post-diagnosis. The number of participants with homogeneous (solid black) and heterogeneous (black border) founder populations are shown at (C) diagnosis and (D) approximately six months post-diagnosis. The number of sequences per participant are shown at (E) diagnosis and (F) approximately six months post-diagnosis.

**S2 Frequency of CRF01_AE consensus and non-consensus amino acids at sieve sites for all (110) participants at diagnosis**


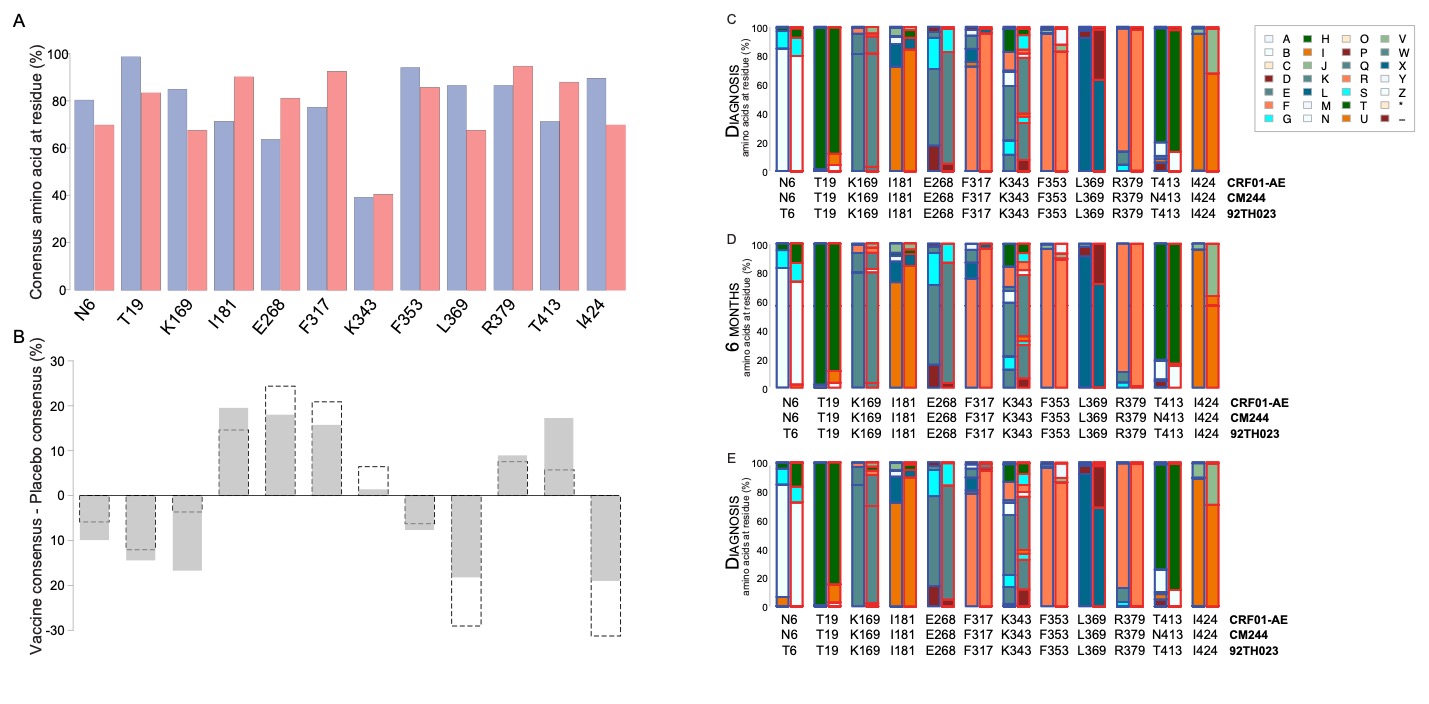


**Figure S2: Frequency of CRF01 AE consensus and non-consensus amino acids at select sites for all (110) participants at diagnosis.** (A) Frequency of consensus amino acids at sieve sites for 66 placebo (blue) and 44 vaccine (red) recipients sampled at diagnosis. (B) Percentage difference between the proportion of consensus amino acids at each VE site between placebo and vaccine recipients at diagnosis. The dashed bars show the differences when only participants sampled at both time points (45 placebo, 28 vaccine) were used. Frequency of different amino acids at VE sites in vaccine and placebo recipients at (C) diagnosis and (D) post-diagnosis. (E) Frequency at diagnosis for all participants sampled at diagnosis (44 vaccine and 66 placebo recipients).

**S3 Amino acid frequency at sieve sites in circulating CRF01_AE sequences**

**
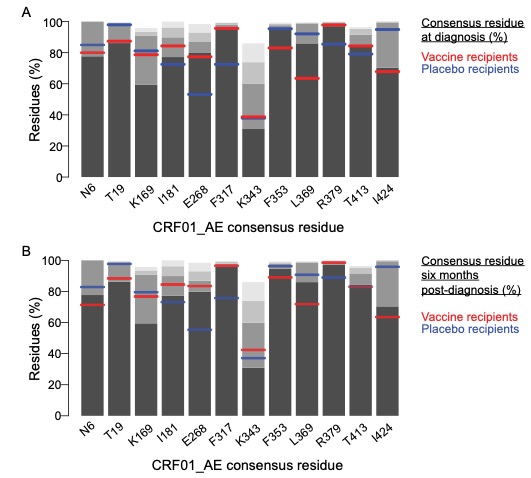
**

**Figure S3: Amino acid frequency at sieve sites in circulating CRF01_AE sequences.** Stacked barplot of the top four most frequently occurring residues at 12 sieve sites in 521 independent circulating CRF01_AE Env sequences (downloaded from the HIV-1 LANL database). (A) The frequency of the consensus residue in circulating sequences is compared to that of the consensus residue at each site in vaccine (red) and placebo (blue) recipients at (A) diagnosis and (B) six months post-diagnosis.

**S4 Schematic of estimating a per-participant Vaccine Response (VR) score**


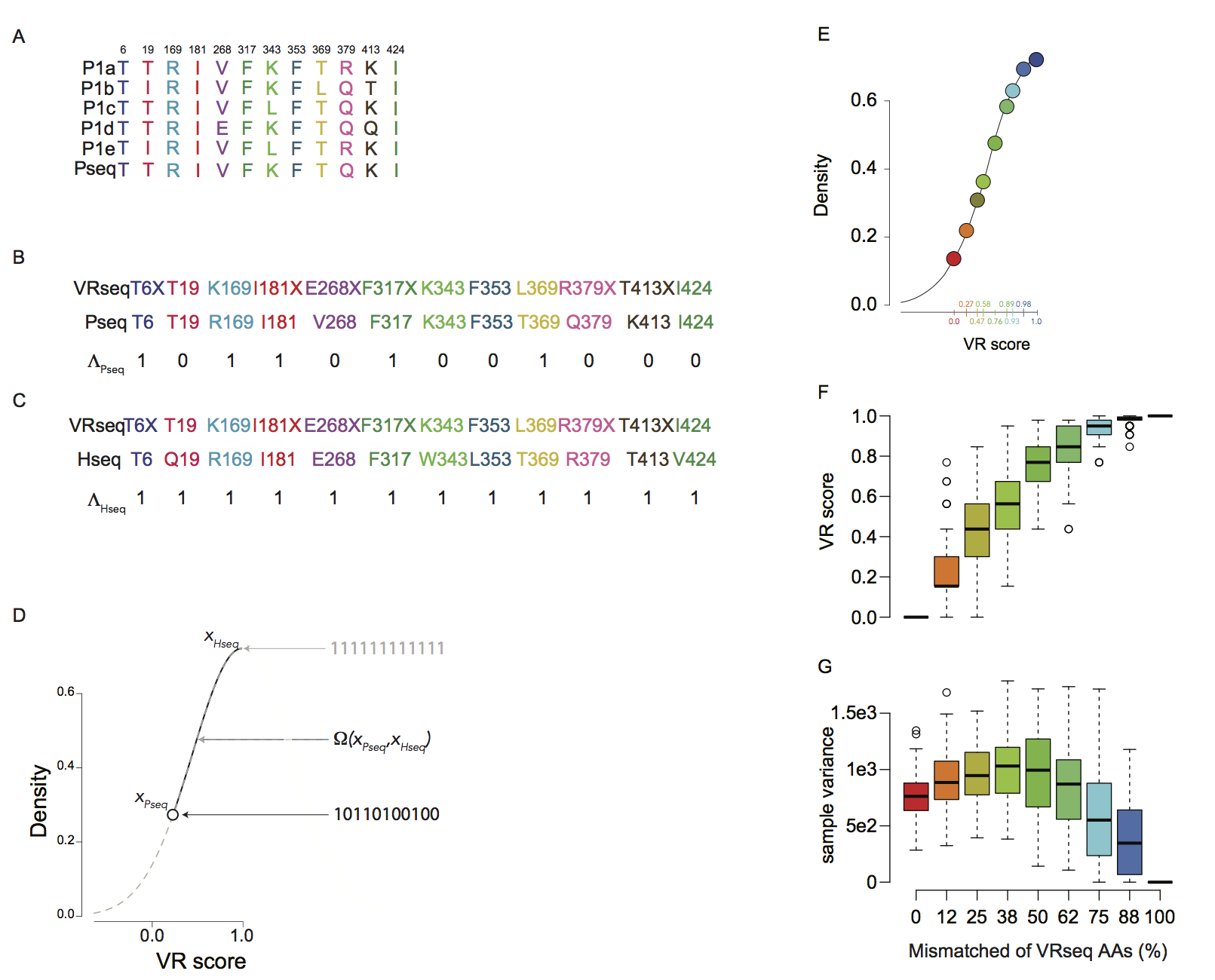


**Figure S4: Schematic of estimating a per-participant Vaccine Response (VR) score.** (A) A majority-rule consensus is inferred for a participant, Pseq. (B) Each Pseq site is counted as 0 if it is identical to the amino acid at that site in the VRseq and 1 otherwise. (C) A hypothetical sequence, Hseq, is created where each amino acid is mismatched with the VRseq. (D) A probability density function (pdf) is computed for Hseq (grey). The position of the mean value of Pseq is interpolated on the pdf. The VR score is estimated as one minus the distance (black and grey segment on the pdf) between the position of the participant, *x_Pseq_*, and the apex of the pdf, *x_Hseq_*. (E-G) Recovering simulated VR scores. (E) Probability density function of a sequence of length 12 and median 1 (grey). The interpolated positions of simulated participants with sequences corresponding to varying vaccine efficacy are shown as filled circles with their VR scores along the x-axis. Boxplot of (F) VR scores and (G) sample variances for participants simulated with sequences corresponding to varying vaccine efficacy. For each proportion, 100 participants were simulated.

**S5 Mutations at sites associated with T cell pressure**


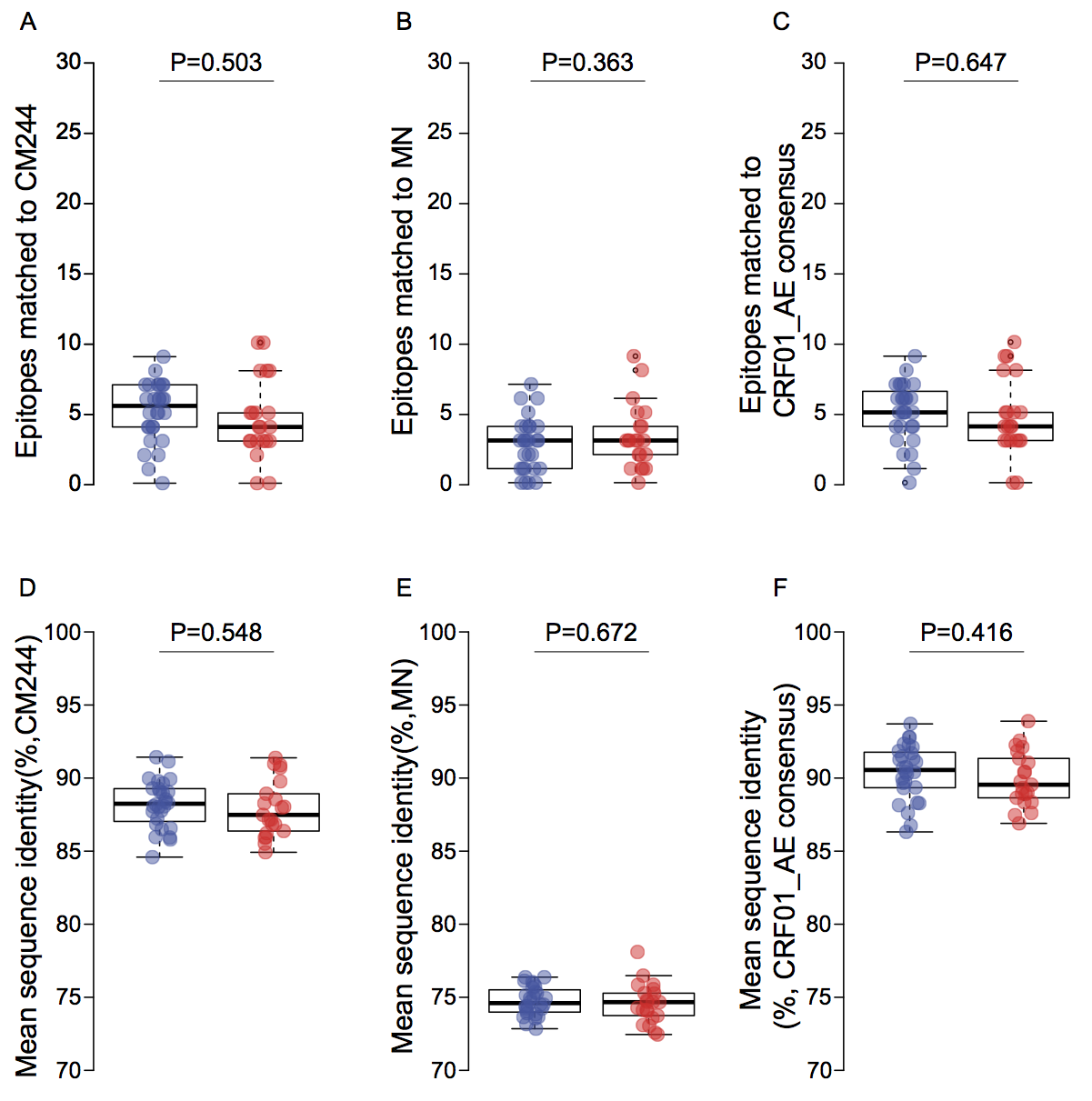


**Figure S5: Mutations at sites associated with T cell pressure.** Number of CTL epitopes matching the vaccine inserts (A) CM244 and (B) MN and the (C) CRF01_AE consensus in vaccine (in red) and placebo recipients at diagnosis. Mean sequence identity between vaccine and placebo recipients and vaccine inserts (D) CM244 and (E) MN and (F) CRF01 AE consensus. P-values for pairwise Student’s t tests are shown above each pair.

**S6 Temporal changes in diversification**


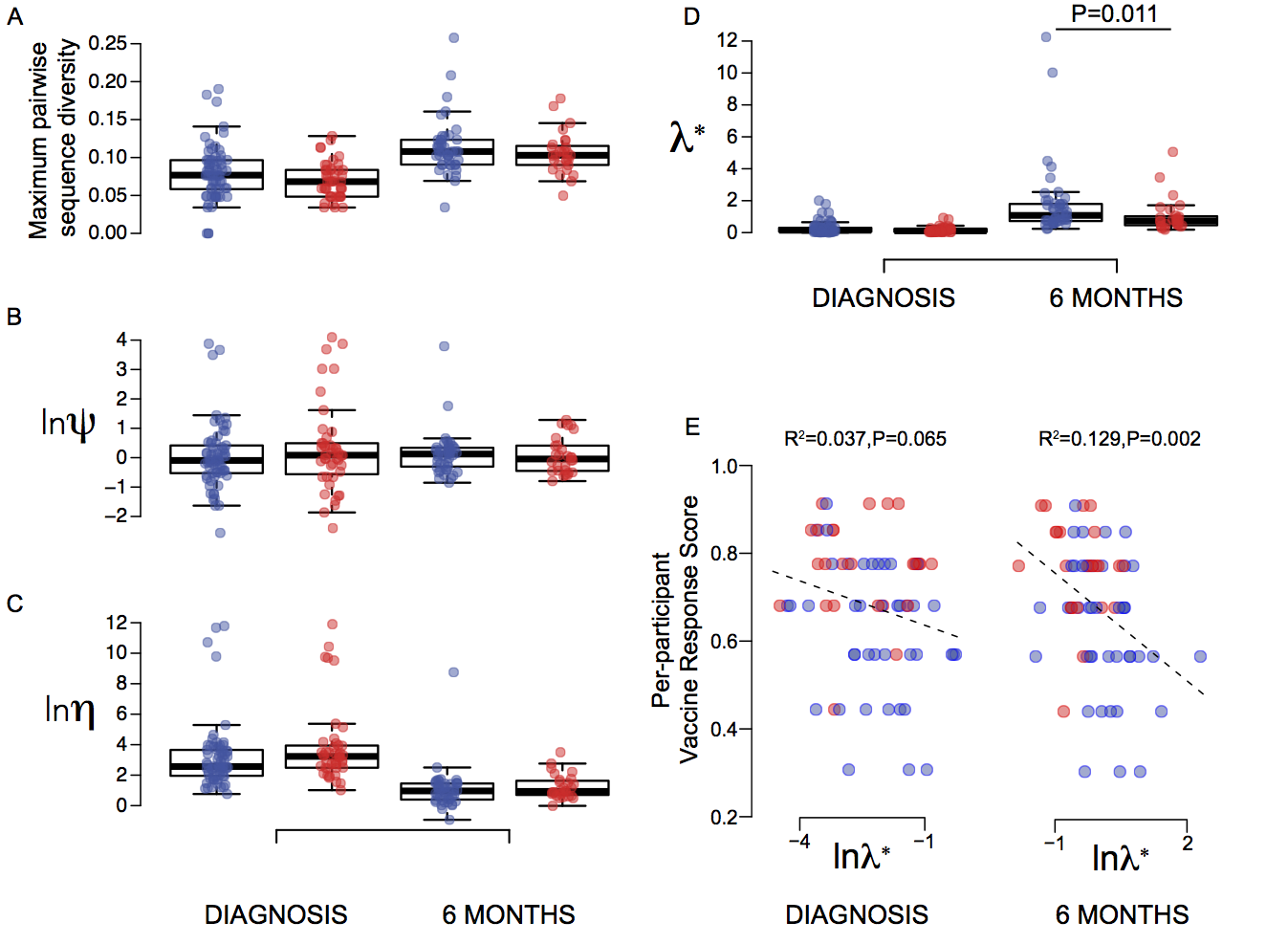


**Figure S6: Temporal changes in diversification.** Boxplots of (A) maximum pairwise sequence diversity and the (B) ln-transformed ψ, (C) ln-transformed η, and (D) λ∗ of spectral density profiles across par- ticipants treated with vaccine (red) and placebo (blue) at diagnosis and six months post-diagnosis. Per-participant VR scores computed on VE sites as a function of ln-transformed l∗ at (E) diagnosis and six months post-diagnosis. (A-D) Significant pairwise differences between treatment groups within the same sampling time are indicated with the associated p-value (Mann-Whitney U test). (E) Best-fit linear regressions are shown (dashed line) along with R^2^ and p-values.

**S7 The effect of founder heterogeneity on l***


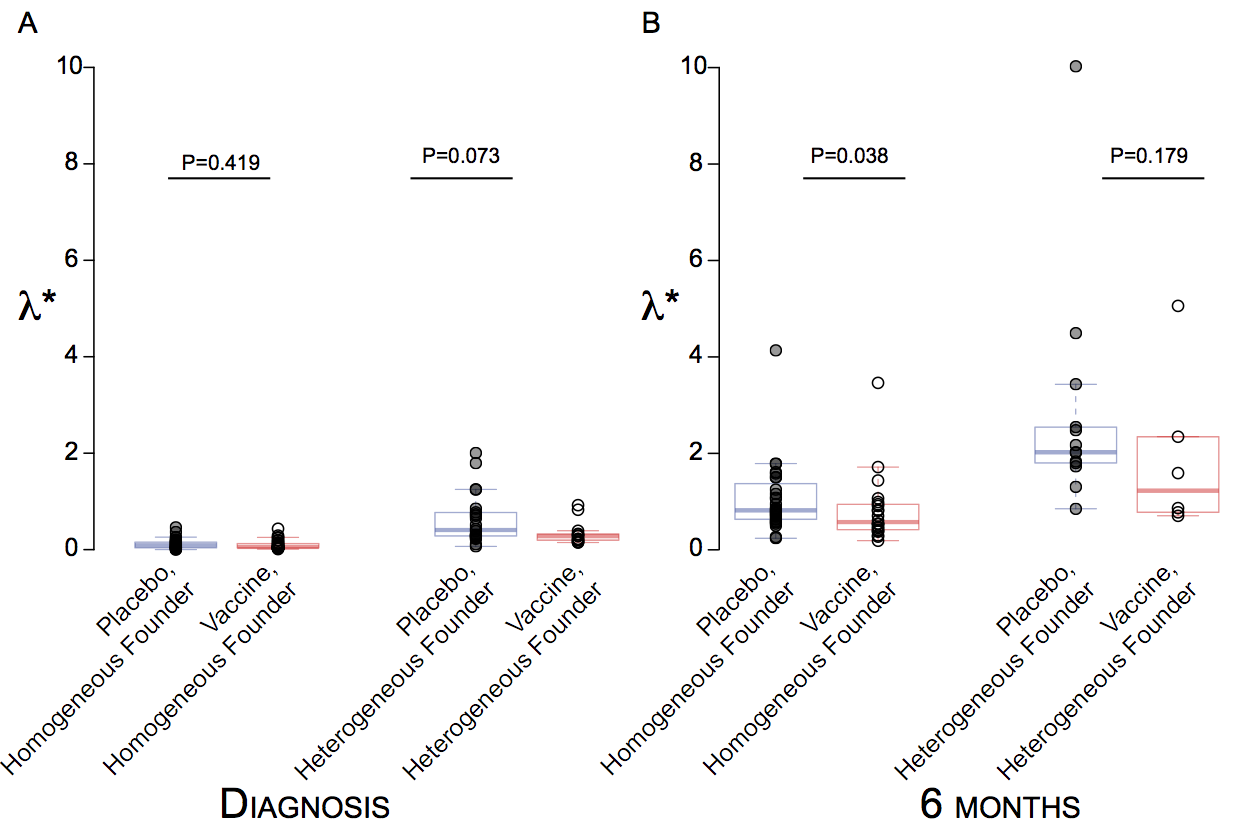


**Figure S7: Effects of founder heterogeneity on l^∗^.** Boxplots of l^∗^ in placebo (blue) and vaccine (red) recipients with homogeneous (open circles) or heterogeneous (closed circles) founders at (A) diagnosis and (B) six months post-diagnosis. P-values for pairwise Mann-Whitney U tests are shown above each pair.

**S8 Phylogenetic space of vaccine and placebo participants at diagnosis and six months later**


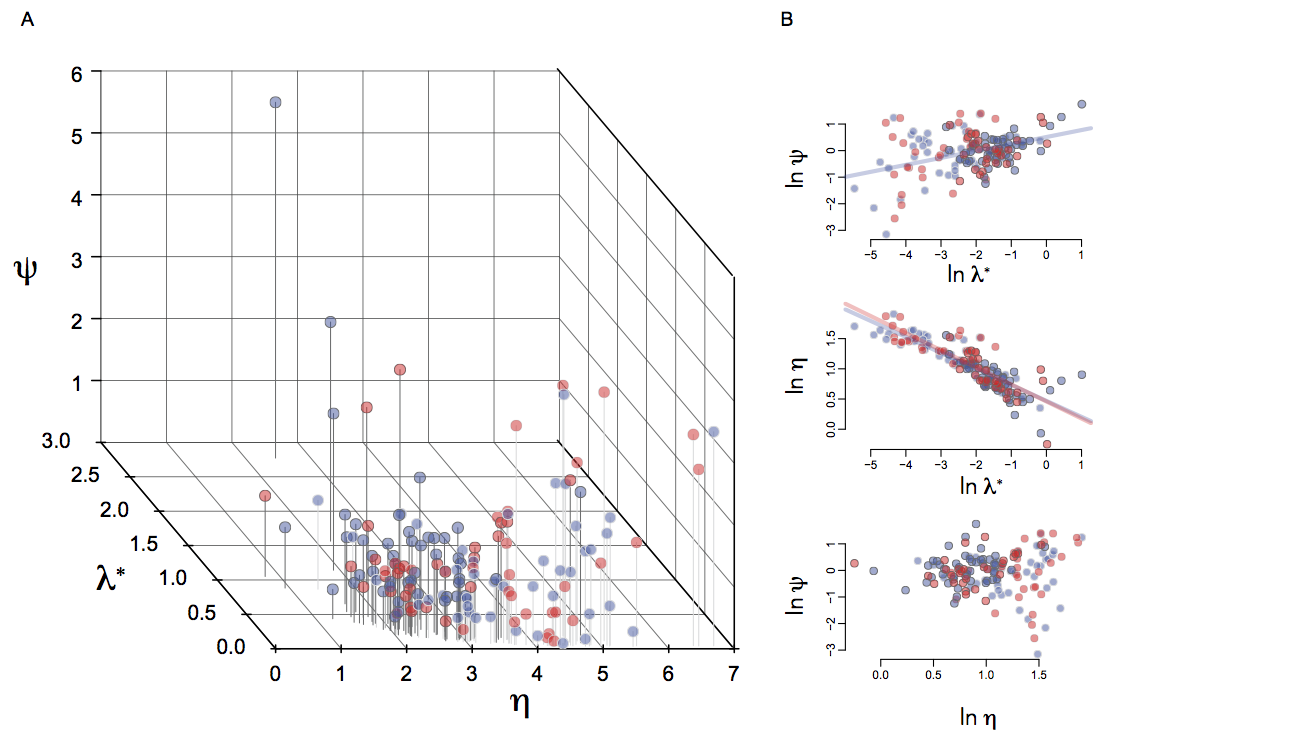


**Figure S8: Phylogenetic space of vaccine and placebo participants at diagnosis and six months later.** (A) Multidimensional plot of spectral density profile summary statistics for 28 vaccine (red) and 45 placebo (blue) recipients sampled at diagnosis (light grey line) and six months later (dark grey line) shows how patterns of HIV-1 diversification traverse phylogenetic space as a function of time rather than treatment, crossing from high-h, low-l^∗^ space to low-h, high-l^∗^ space. (B) Scatterplots of spectral density profile summary statistics at both sampling times show that y increases as a function of l^∗^ for placebo (R^2^ = 0.178, P = 2.30e − 5) but not for vaccine (R^2^ = 0.079, P = 0.061) recipients; η decreases with statistically indistinguishable slopes as a function of l^∗^ for placebo (R^2^ = 0.736, P < 1e − 6) and vaccine (R^2^ = 0.667, P < 1e − 6) recipients; and that there is no significant relationship between h and y for either treatment group (R^2^ < 0.01,P > 0.22). Regression slopes are only shown when significant.

**S9 Temporal changes in sequence diversity across hypervariable domains**


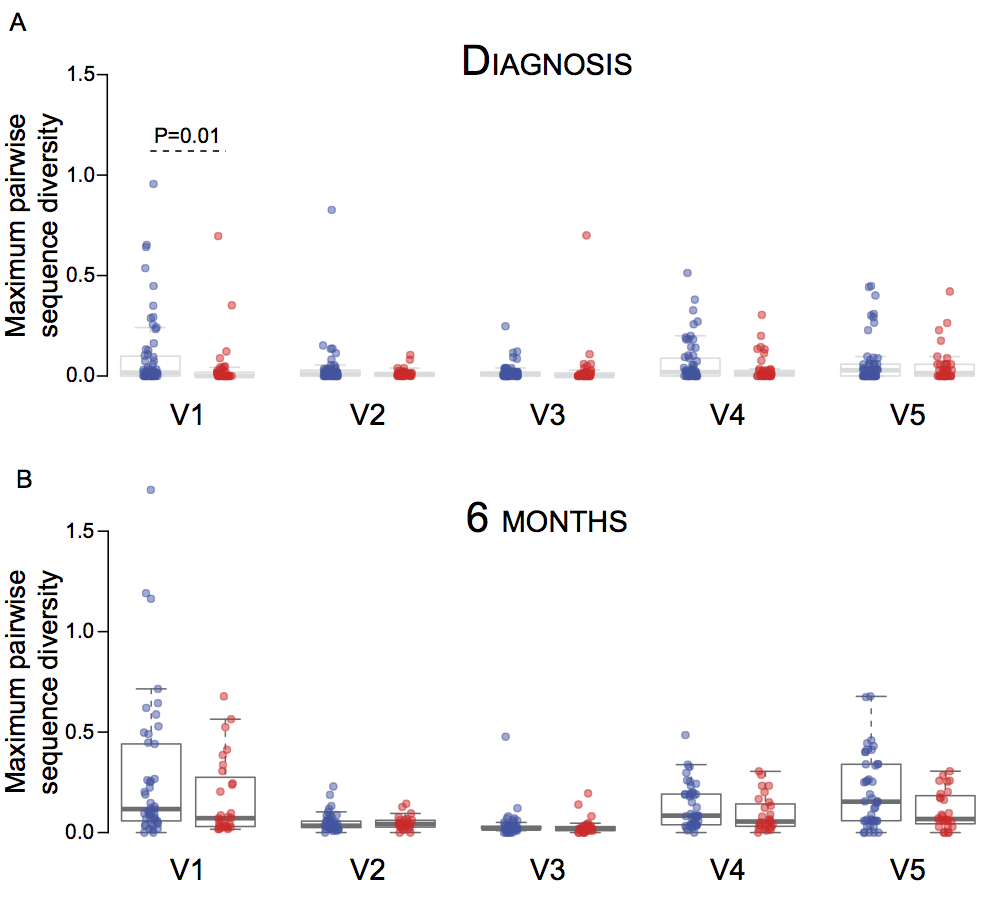


**Figure S9: Temporal changes in sequence diversity across hypervariable domains.** Boxplots of maximum pairwise sequence diversity restricted to each hypervariable domain across treatment groups at (A) diagnosis and (B) six months post-diagnosis. Significant pairwise differences between vaccine (red) and placebo (blue) groups within the same sampling time are indicated with the p-value (Mann Whitney U test). Pairwise differences within treatment groups across sampling times are significant for each hypervariable domain (P < 2.8e − 3).

**S10 The effect of founder heterogeneity on pervasive selection**


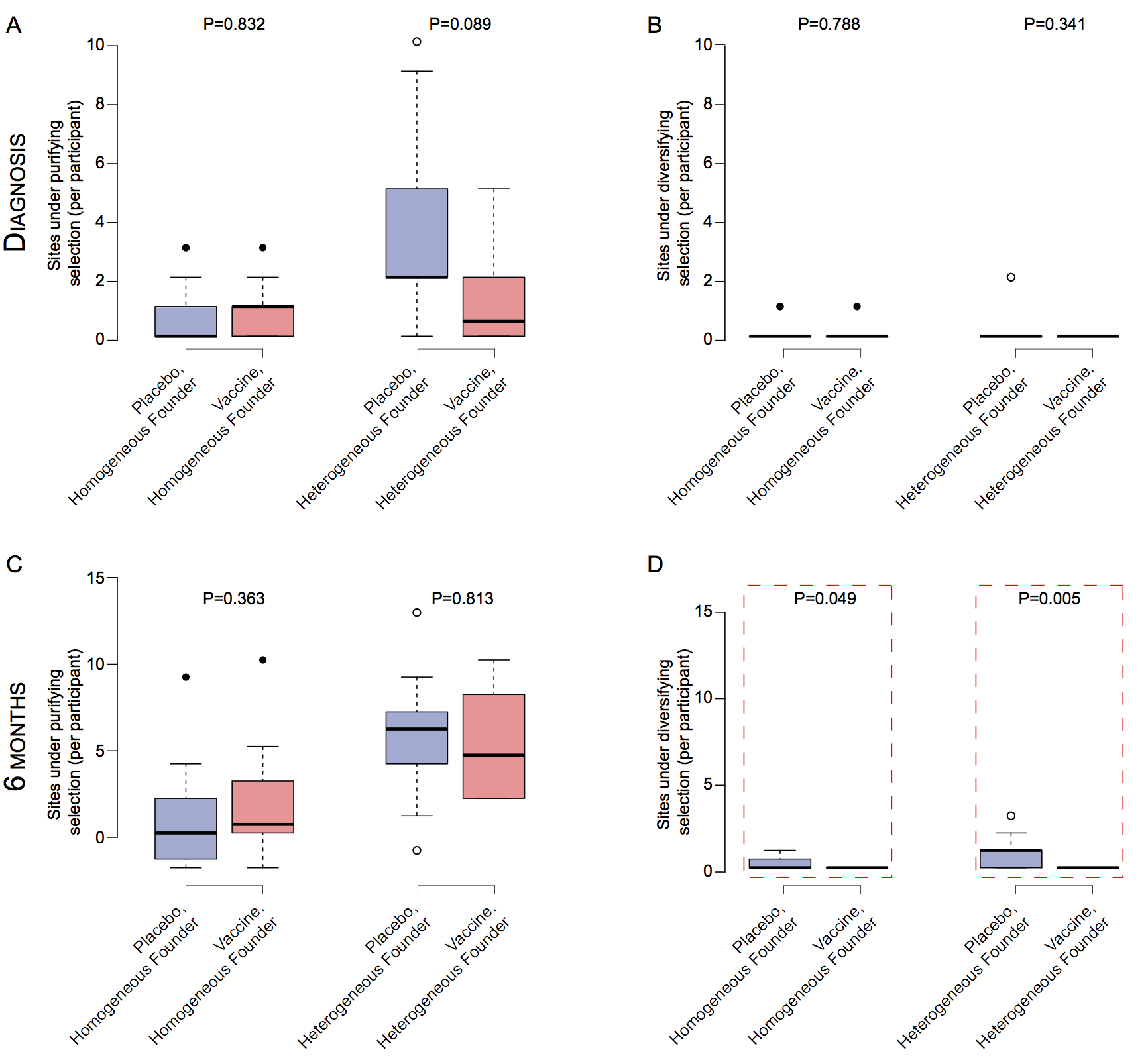


**Figure S10: The effect of founder heterogeneity on pervasive selection.** Boxplots of the number of sites under selection per participant between treatment groups with homogeneous (closed circle) or heterogeneous (open circle) founders. At diagnosis, (A) purifying sites and (B) diversifying sites. Six months post-diagnosis, (C) purifying sites and (D) diversifying sites. P-values for pairwise comparisons are indicated (Mann Whitney U tests). Comparisons with significant p-values are highlighted in dashed red boxes.

**S11 Per-site pervasive selection**


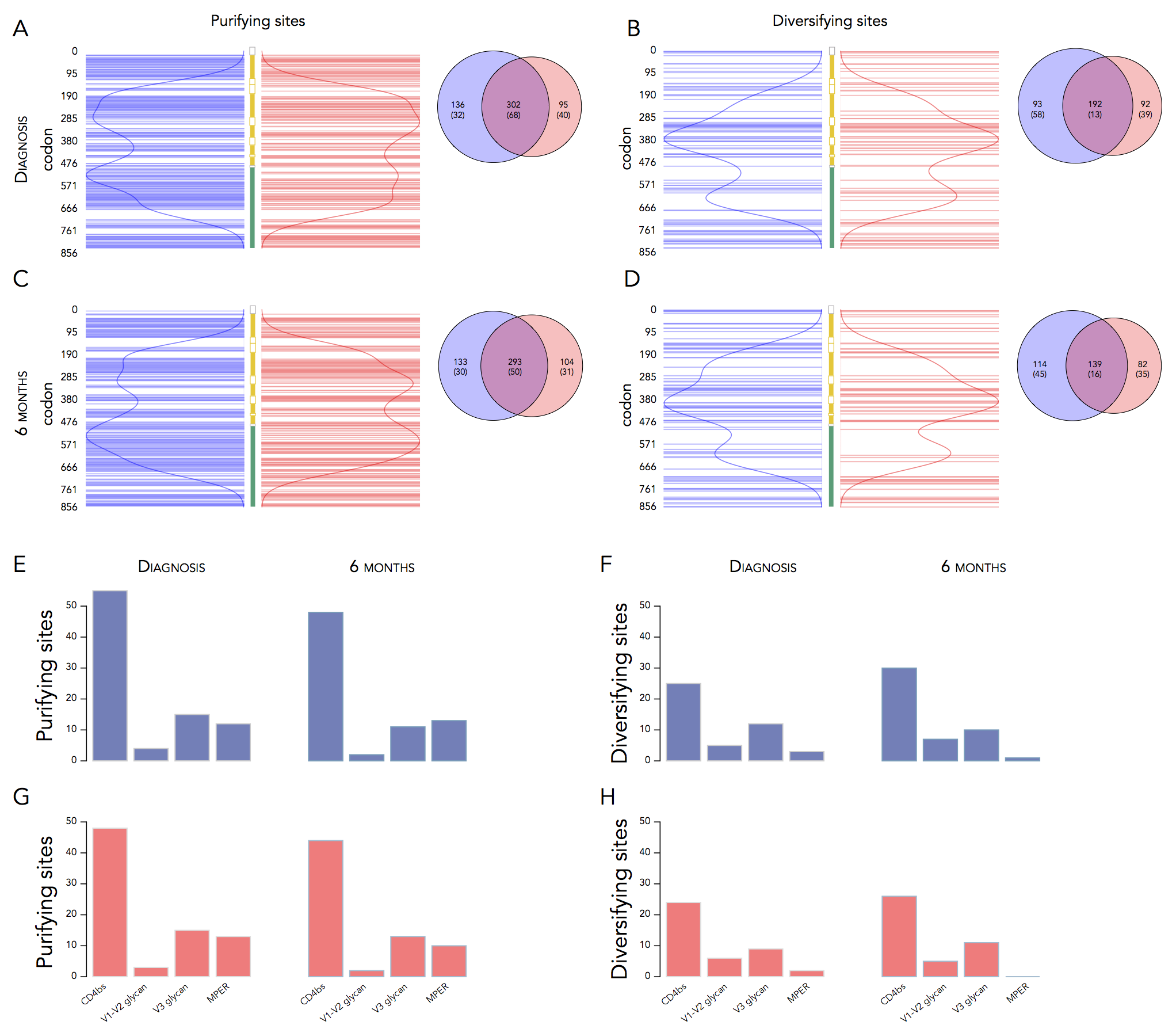


**Figure S11: Per-site pervasive selection.** Barcode plots of (A,C) purifying and (B,D) diversifying sites in participants treated with vaccine (red) and placebo (blue) at (A,B) diagnosis and (C,D) six months post-diagnosis. Sites across all jackknifed alignments are shown in (C,D). Barcode plots are overlaid with density plots. Along the y-axis, a cartoon of the HIV-1 *env* gene is shown depicting gp120 (closed yellow), gp41 (closed green), signal peptides (open grey), and hypervariable do- mains (open yellow). Codons correspond to HXB2 numbering. Venn diagrams of codons under purifying and diversifying selection show the number of sites under selection across all participants (parenthetical numbers refer to contact sites only). Median values are shown in Venn diagrams at six months post-diagnosis. Barplots of the number of purifying and diversifying sites under selection at known antibody contact sites in placebo (E,F) and vaccine (G,H) recipients at diagnosis and six months post-diagnosis.

**S12 The effect of neutralization breadth on pervasive selection**


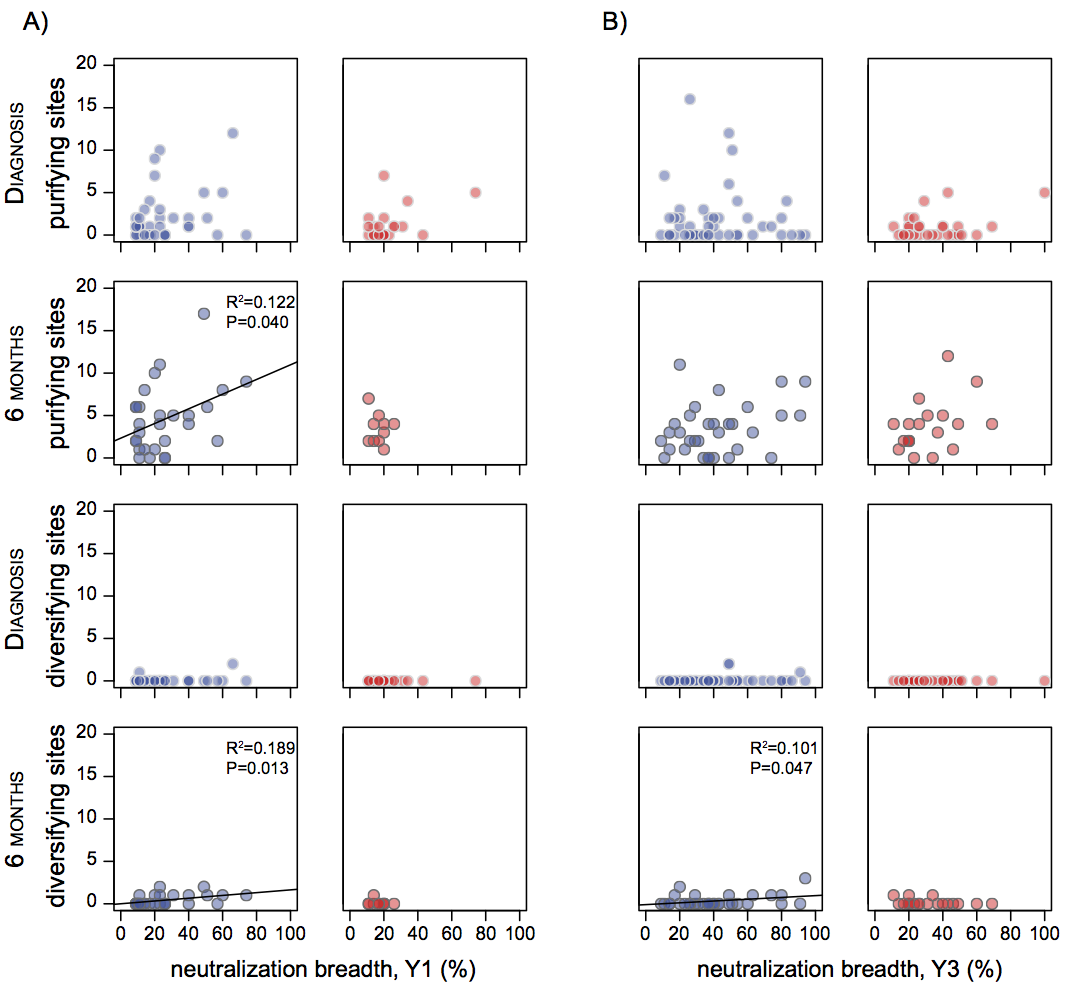


**Figure S12: The effect of neutralization breadth on pervasive selection.** Scatterplots of the number of purifying and diversifying sites under selection per participant as a function of neutralization breadth measured at (A) one year and (B) three years since diagnosis for vaccine (red fill) and placebo (blue fill) recipients at diagnosis (light grey border) and six months post-diagnosis (dark grey border). Regression slopes and R2 values are shown for regression coefficients significant at P < 0.05.
